# Pulling back the mitochondria’s iron curtain

**DOI:** 10.1038/s44324-024-00045-y

**Published:** 2025-03-04

**Authors:** Shani Ben Zichri- David, Liraz Shkuri, Tslil Ast

**Affiliations:** Department of Biomolecular Sciences, Weizmann Institute of Science, Rehovot, 7610001 Israel

**Keywords:** Biochemistry, Cell biology, Metabolic pathways

## Abstract

Mitochondrial functionality and cellular iron homeostasis are closely intertwined. Mitochondria are biosynthetic hubs for essential iron cofactors such as iron-sulfur (Fe-S) clusters and heme. These cofactors, in turn, enable key mitochondrial pathways, such as energy and metabolite production. Mishandling of mitochondrial iron is associated with a spectrum of human pathologies ranging from rare genetic disorders to common conditions. Here, we review mitochondrial iron utilization and its intersection with disease.

## Introduction

Iron played a critical role in the emergence and establishment of life on earth. In the metal-rich anaerobic oceans where early life evolved^1^, iron’s wide redox potential likely enabled rudimentary metabolism, electron transport, and energy transfer^2,3^. Shaped by these origins, modern lifeforms also heavily rely on iron cofactors to catalyze fundamental reactions. Conservative estimates indicate that hundreds of human proteins bind iron^4^, facilitating DNA replication and repair, gene expression, oxygen transport, and beyond. These proteins either engage an iron ion directly or bind to an iron complex as an iron-sulfur (Fe-S) cluster or heme. While these iron cofactors are tightly “tuned” by their binding proteins to create selective reduction potential windows tailored to their biological functions, collectively, they span a remarkable range from −770 to +440 mV^5^. Being so customizable, these cofactors enable a wide spectrum of redox reactions.

**Fig. 1:**
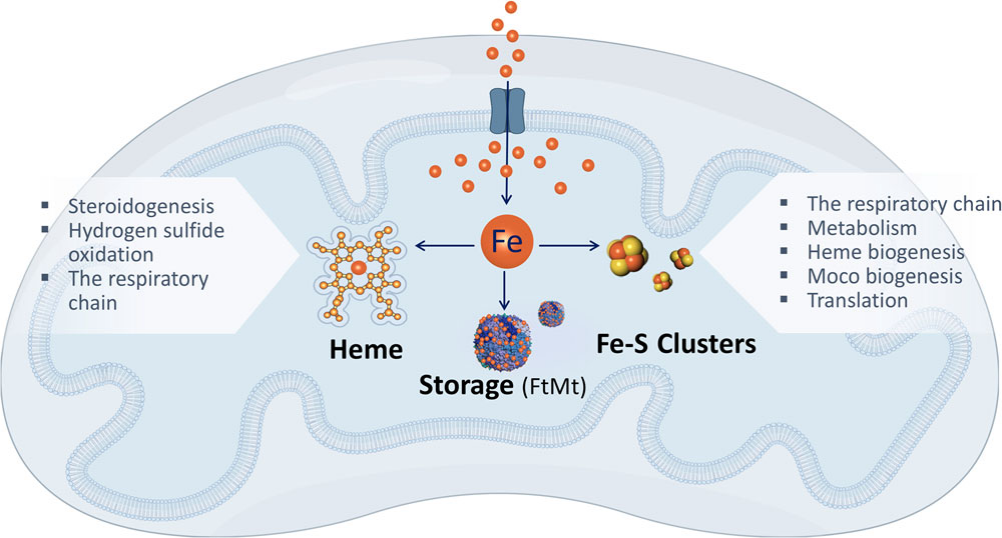
Overview of mitochondrial iron utilization. Upon entering the mitochondrial matrix, iron can be channeled to three pathways: heme biosynthesis, iron-sulfur (Fe-S) cluster biogenesis, or storage in mitochondrial ferritin (FtMt). The cofactors produced by mitochondrial iron, namely Fe-S clusters or heme, subsequently support various essential mitochondrial tasks.

Within the eukaryotic cell, the mitochondrion stands out both for its notable contributions to iron cofactor biosynthesis and utilization. First, both heme and Fe-S cluster biosynthesis occurs, in part, in mitochondria. Comparative evolutionary analysis indicates that the latter is among the most conserved mitochondrial-residing pathways^6^. Second, the relative engagement of iron cofactors within the mitochondrial proteome is ~3.5-fold higher than the total proteome^4^. These cofactors are prominent in the electron transport chain, mitochondrial anabolic and catabolic pathways, and mitochondrial translation apparatus. In addition, mitochondria can also take part in iron storage, by virtue of mitochondrial ferritin (FTMT). Thus, mitochondria are at the crossroads of cellular iron metabolism and engagement. Several excellent recent reviews have discussed how cells acquire iron, and how this metal is subsequently transferred to the mitochondrial matrix^7,8^. Here we will highlight how iron is metabolized or utilized in the mitochondria to support the biology of this multifaceted organelle (Fig. 1).

## Mitochondrial iron handling

### Iron-sulfur cluster biogenesis

Made up of different combinations of inorganic iron and sulfur, Fe-S clusters can exist in numerous forms, the most common being [2Fe-2S], [3Fe-4S], and [4Fe-4S] clusters^9^. As electrons are delocalized over both iron and sulfur ions in the cluster^10^, Fe-S clusters are highly suited for electron transfer, redox sensing, or catalysis. In addition, these versatile cofactors can also take part in sulfur donation reactions and have been shown to contribute to the structural stability of the proteins into which they are integrated.

**Fig. 2:**
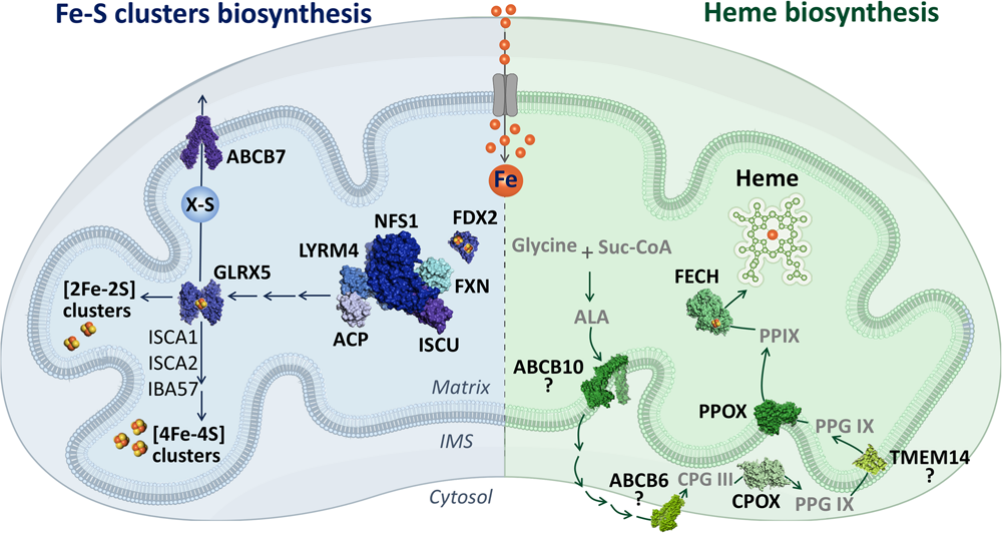
Simplified overview of Fe-S cluster and heme biosynthesis. Mitochondrial iron-sulfur (Fe-S) cluster biogenesis (left) is initiated by a protein complex, comprised of key proteins such as the ISCU scaffold, the cysteine desulfurase NFS1, and the allosteric activator FXN. This complex generates 2Fe-2S clusters, which are relayed to GLRX5. Subsequent machinery, comprising ISCA1, ISCA2, and IBA57, can build on these clusters to form 4Fe-4S clusters. Alternatively, some intermediate can be handed off to the cytosol via the inner membrane transporter ABCB7. Concurrently, heme biosynthesis (right) begins with the condensation of glycine and succinyl-CoA (Suc-CoA) to form 5-aminolevulinic acid (ALA). ALA is exported to the cytosol, potentially by ABCB10, where it undergoes further processing before being re-imported into the mitochondrial inner membrane space, possibly via ABCB6. Within this compartment, CPOX catalyzes the formation of protoporphyrin IX (PPIX), which is then transported to the mitochondrial matrix and converted to heme by FECH. PDB IDs for protein structures: ABCB7 (7VGF), GLRX5 (2WUL), FDX2 (2Y5C), LTRM4-NFS1-FXN-ISCU-ACP (8PK8), ABCB10 (7Y49), FECH (1HRK), PPOX (3NKS), CPOX (2AEX), ABCB6 (7DNY), and TMEM14 (2LOR).

While Fe-S cluster formation was originally thought to occur spontaneously within cells^11–13^, it is now appreciated that clusters must be actively synthesized and relayed to the proteins that require them^14–16^ (Fig. 2 left). The first step of biogenesis involves the iron-sulfur cluster (ISC) machinery, which catalyzes the synthesis of a [2Fe-2S] cluster from ferrous iron, sulfur (provided by cysteine), and electrons. The cluster is built upon the scaffold protein, ISCU, and relies upon the rate-limiting activity of the cysteine desulfurase complex NFS1-LYRM4-NDUFAB1. Cluster biogenesis also demands electrons, provided by ferredoxin 2 (FDX2), and is allosterically activated by frataxin (FXN). Once the initial [2Fe-2S] cluster has been generated, it is transferred to the glutaredoxin 5 (GLRX5) dimer with the help of the chaperone/co-chaperone pair HSPA9/HSC20. This process requires energy supplied by ATP hydrolysis carried out by HSPA9^17^. At this step, an intermediate is generated that can be relayed to the cytosol via the inner mitochondrial membrane (IMM) transporter ABCB7, supporting the formation of cytosolic and nuclear Fe-S clusters. Of note, several reports have indicated that the ISC pathway may be simultaneously active in the cytosol^18–20^. Finally, the generation of mitochondrial [4Fe-4S] clusters is carried out by ISCA1 and ISCA2, which reductively couple two [2Fe-2S] clusters^10,21^. This reaction also requires IBA57, whose mechanistic role remains poorly defined, and electron transfer from FDX2. Together, this complex multistep pathway ensures the controlled production and relay of labile clusters to their various acceptor proteins.

### Heme biosynthesis

Heme, an iron-containing tetrapyrrole^22^, can unlock diverse abilities including oxygen binding^23^, electron transport^24^, and enzymatic catalysis. Thus, heme is essential for virtually all aerobic lifeforms. Unlike the case for Fe-S clusters, cells can take up heme from their environment. However, the relative contributions of endogenous vs. exogenous supply to organellar and cellular heme pools remains an area of active investigation.

Heme biosynthesis in most metazoans involves a coordinated sequence of eight enzymatic reactions, which traverse the mitochondria and cytosol (Fig. 2 right). A notable exception to this biosynthetic capability are the nematode *Caenorhabditis elegans* and other parasitic helminths, which are heme auxotrophs^25^. However, the majority of animals are capable of synthesizing heme de novo from basic substrates: ferrous iron, glycine, and succinyl-CoA. The mitochondrial enzyme ALA synthase (ALAS) initiates the rate-limiting condensation of succinyl-coenzyme A and glycine to form 5-aminolevulinate (ALA)^26^. Once ALA is generated, it must be exported to the cytosol via an unidentified transporter, although there are indications that ABCB10 might be involved in this process^27,28^. In the cytosol, ALA is converted into coproporphyrinogen III (CPG III) through a series of four reactions. The final steps of heme biosynthesis occur back in mitochondria. First, CPG III is transported into the intermembrane space, possibly by ABCB6^29^, where it is oxidized to protoporphyrinogen IX (PPG IX) by coproporphyrinogen III oxidase (CPOX). This is notable, as there are very few metabolic reactions that are carried out in the intermembrane space. Next, PPG IX is transported into the mitochondrial matrix, potentially via the transmembrane protein TMEM14C^30^, and oxidized to form protoporphyrin IX (PPIX) by the IMM enzyme protoporphyrinogen oxidase (PPOX)^22^. The final mitochondrial step involves the insertion of ferrous iron into the protoporphyrin ring to form the heme group by ferrochelatase (FECH)^31^. Thus, the complex heme biosynthesis pathway both starts and ends in mitochondria, although the benefits conferred by this elaborate spatial segregation remain unclear.

### Mitochondrial iron sequestration

Mitochondria from varied sources have been shown to contain a sizeable 20–50% of the total cellular iron content^32,33^. Excess iron can be stored in the mitochondrial form of ferritin (FTMT), a homopolymer that is structurally and functionally similar to the cytosolic ferritin heavy chain^34^. By sequestering free iron, FTMT may limit the iron-driven production of reactive hydroxyl radicals via the Fenton reaction^35^. Notably, FTMT knockout mice are viable and display no significant defects in systemic iron metabolism under baseline conditions^36^, but have been shown to be susceptible to oxidative or neurotoxic insults^37–40^. Further hinting at a role in oxidative regulation, FTMT is primarily expressed in the testis^41^, rather than iron-rich tissues such as the liver and spleen. Taken together, these data suggest that the primary function of FTMT may be regulatory and protective, rather than a constant buffer of mitochondrial iron pools^42,43^.

## Iron-dependent mitochondrial pathways

### Fe-S cluster-dependent processes

#### The respiratory chain

The mitochondrial respiratory chain is a central redox hub of the eukaryotic cell. While classically depicted as four macromolecular IMM complexes working in a linear fashion to couple electron transfer with proton pumping, the respiratory chain is, in fact, a dynamic sink for many cellular redox reactions. In metazoans, these include de novo pyrimidine biosynthesis, sulfide metabolism, fatty-acid oxidation, amino acid metabolism, and glycolysis.

**Fig. 3:**
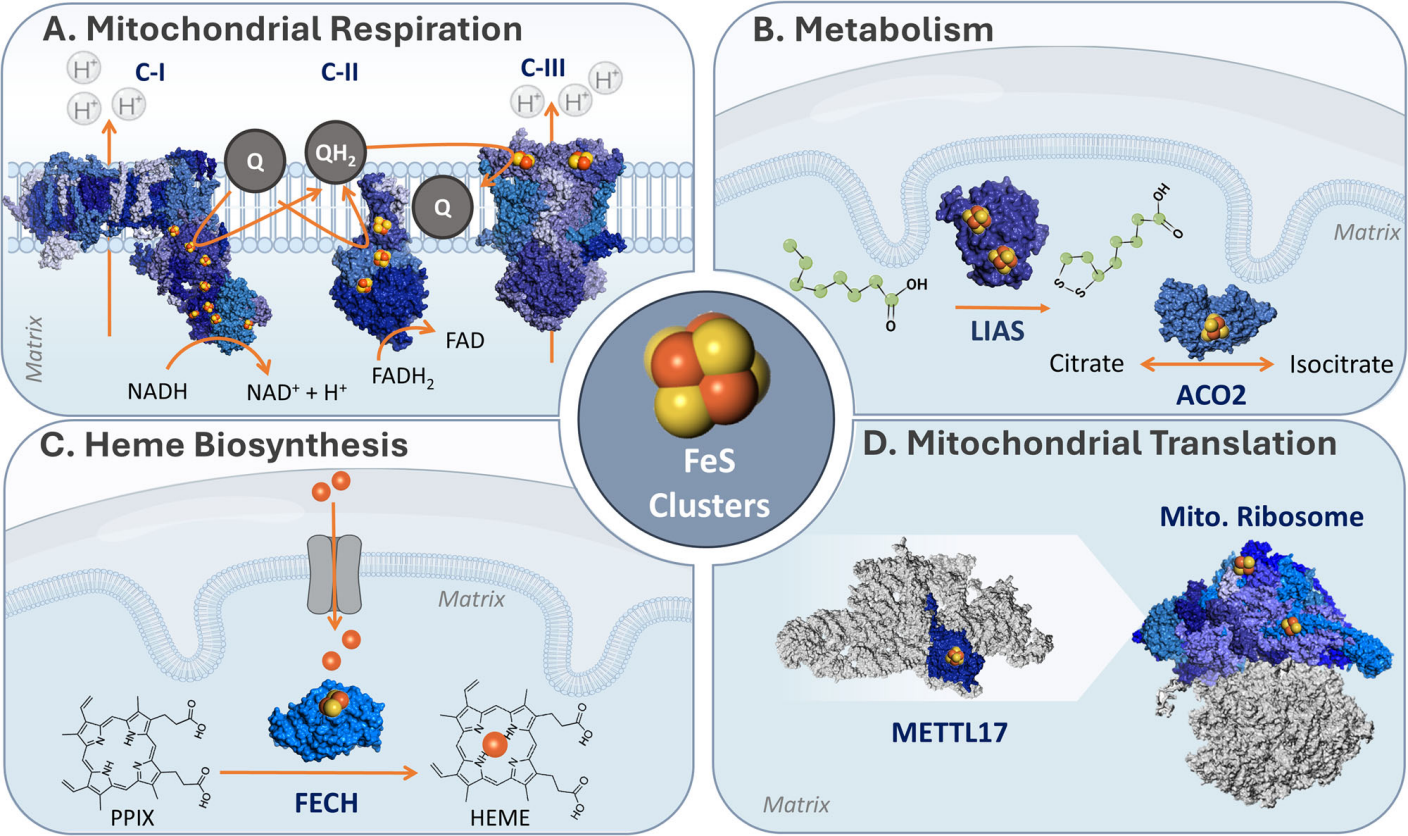
Overview of central Fe-S cluster pathways in the mitochondria. **A** Mitochondrial respiration. Fe-S clusters take part in electron transfer in complexes I, II, and III in the respiratory chain, found in the inner mitochondrial membrane. **B** Mitochondrial metabolism. (i) Lipoic acid synthesis requires LIAS, which catalyzes the insertion of two sulfur atoms into a protein-bound octanoyl chain; (ii) The TCA enzyme mitochondrial aconitase, which reversibly converts citrate to isocitrate. **C** Heme biosynthesis. Fe-S clusters are required in the second and final steps of heme biosynthesis. The last is present in the mitochondria, whereby FECH catalyzes the insertion of a ferrous iron into the protoporphyrin ring. **D** Mitochondrial translation. The biogenesis and structure of the mitochondrial ribosome require Fe-S clusters. The late-stage small mitoribosome subunit assembly factor, METTL17, binds an 4Fe-4S cluster essential for its stability. In addition, two Fe-S clusters are found in the small subunit of the mitoribosome, where they stabilize protein-protein interaction interfaces. PDB IDs for protein structures: Complex I(4WZE), Complex II (8GS8), Complex III (5XTE), LIAS (5EXK), ACO2 (5ACN), FECH (1HRK), METTL17 (8OM2), and Mitoribosome (6QZP and 7P2E).

Consequently, it is not surprising that the respiratory chain is rich in Fe-S clusters that help channel electrons (Fig. 3A)^44,45^. Specifically, the ‘early’ steps of the respiratory chain that feed electrons to coenzyme Q (CoQ)- Complexes I and II- are abundant in Fe-S clusters. Complex I (NADH: ubiquinone oxidoreductase) is the primary entry point of electrons into the respiratory chain, passing reducing equivalents from NADH to CoQ^46^. To carry out this reductive reaction, Complex I binds flavin and, depending on the organism, up to ten Fe-S clusters^47^. Working as an “electron wire”, these Fe-S clusters connect the two substrate-binding sites that lay over 90 Ȧ apart^48,49^. Complex II (succinate dehydrogenase) works at the interface of the tricarboxylic acid (TCA) cycle and the respiratory chain. While not contributing to proton pumping, complex II oxidizes succinate to fumarate and transfers these electrons to CoQ. Three Fe-S clusters, all bound to the subunit SDHB, are essential for this activity^50^. Lastly, complex III (cytochrome bc1) accepts electrons relayed via CoQH_2_ and relays them to cytochrome c. A [2Fe-2S] cluster in this complex is coordinated by the Rieske iron–sulfur protein (UQCRFS1), and directly accepts electrons from CoQH_2_. This represents the first and rate-limiting step of the Q cycle, in which these electrons will be subsequently relayed to cytochrome c (i.e. the high-potential redox chain). In parallel, electrons produced via fatty-acid oxidation and amino acid catabolism are also channeled to CoQ via an Fe-S cluster-binding protein. Specifically, the [4Fe-4S] cluster-binding electron transfer flavoprotein dehydrogenase (ETFDH) aids in transferring electrons from multiple mitochondrial acyl-CoA dehydrogenases to CoQ^51^. In this way, multiple reductive sources “feed” electrons to the respiratory chain via Fe-S clusters.

Mutations in the mitochondrial Fe-S cluster biosynthesis or relay machineries have been shown to dramatically blunt respiration in multiple model systems, most notably affecting complex I and II stability. These clusters are also labile in the face of environmental insults, such as oxygen toxicity^52^. Recent work has highlighted an auxiliary Fe-S binding protein which may buffer complex I Fe-S cluster levels- the CDGSH iron-sulfur domain 3 protein (CISD3 or MiNT). Found in the mitochondrial matrix, CISD3 belongs to the highly conserved [2Fe-2S]-binding NEET protein family. Fe-S clusters bound to NEET proteins can participate in electron transfer reactions^53,54^, but are bound by a labile and redox-active binding pocket. This later trait supports cluster transfer between NEET and acceptor proteins. CISD3 has been shown to engage with and donate its Fe-S cluster to Complex I subunit NDUFV2^55^. Interestingly, NDUFV2 binds the N1a cluster in complex I, which is not part of the “electron wire”. Rather, the N1a cluster has been suggested to prevent excessive ROS formation or to be required for Complex I assembly and stability.

In summary, Fe-S cluster are crucial for mitochondrial respiration, enabling ATP synthesis, redox rebalancing (NADH, CoQ), and the mitochondrial membrane potential that drives transport of proteins and metabolites across the MIM.

#### Metabolism

Fe-S clusters are woven throughout various metabolic mitochondrial pathways (Fig. 3B), affecting this organelle’s capacity for anabolism and catabolism in distinct ways. While some of these clusters take part in electron transfer reactions, others enable alternative functions such as catalysis, regulation, and sulfur donation.

##### TCA cycle and its cofactors

The TCA cycle, a core axis of mitochondrial metabolism, relies on Fe-S clusters both directly and indirectly at several different nodes (Fig. 3B). First, the TCA enzyme mitochondrial aconitase (ACO2), which catalyzes the reversible isomerization of citrate to isocitrate^56^, has a [4Fe-4S] cluster at its substrate-binding site. ACO2 exemplifies the catalytic capacity of clusters- this [4Fe-4S] cluster acts as a Lewis acid, by virtue of a Fe ion in the cluster that is not bound by ACO2^57^. Second, Complex II, highlighted in the section “The respiratory chain”, converts succinate to fumarate through the action of its three Fe-S clusters. Finally, two key dehydrogenases associated with the TCA cycle require a cofactor generated via an Fe-S cluster-dependent reaction. Specifically, the catalytic activities of pyruvate dehydrogenase and α-ketoglutarate dehydrogenase are critically dependent on the production of lipoic acid, a straight-chain fatty-acid containing two sulfhydryl groups. The biogenesis of this cofactor necessitates the involvement of two Fe-S cluster-binding proteins: lipoic acid synthase (LIAS) and ferredoxin 1 (FDX1). LIAS binds two [4Fe-4S] clusters—the first mediates a radical SAM reaction that activates the aliphatic chain for sulfur attachment, while the second acts as a sulfur donor and must be replenished after each reaction^58^. However, the source of the electron that jumpstarts the radical SAM reaction on LIAS has remained unclear for some time. Several groups have recently shown that FDX1, apart from its previously established roles in processes such as steroidogenesis^57^, is also vital for lipoic acid biosynthesis^59–61^. By contributing electrons for the radical SAM reaction, FDX1 plays a crucial role in maintaining the TCA cycle. Altogether, by enabling the TCA cycle, Fe-S clusters take part in the controlled combustion of nutrients and supply metabolic precursors for nucleotide, lipid, and protein biogenesis.

##### Mitochondrial glutathione balance

The small peptide glutathione plays a principal role in cellular antioxidant defense. Readily transitioning between its reduced monomeric and oxidized dimeric forms (GSH vs. GSSG), glutathione can neutralize reactive oxygen species both directly and indirectly (via antioxidant enzymes). This buffering capacity is particularly pertinent in the mitochondria, where the respiratory chain can produce the highly reactive O_2_.^–^ oxidant^62^. However, glutathione is generated in the cytosol, and while had long been known that up to 15% of cellular glutathione is found in the mitochondria^63^, how it passes the IMM remained unresolved until late. Two independent studies revealed that GSH is transported into the mitochondrial matrix via the solute carrier family 25 paralogues- SLC25A39 and SLC25A40^64,65^. While SLC25A40 appears to be constitutively expressed, SLC25A39 protein is strongly upregulated upon GSH depletion. How is this autoregulation mechanism achieved? Further work into SLC25A39 has revealed that it binds an [2Fe-2S] cluster within a cytosolic loop. Importantly, this cluster can be competed out of SLC25A39 with GSH, destabilizing the protein under high GSH conditions^66,67^. Thus, Fe-S cluster binding in SLC25A39 highlights the metabolite-sensing potential of these cofactors.

#### Heme biogenesis

Heme and Fe-S clusters are tightly linked- the biosynthesis of the former requires the latter. Specifically, both the second^68^ and last^69^ steps of heme biosynthesis are catalyzed by Fe-S cluster proteins. Focusing on the terminal mitochondrial reaction, it has been shown that FECH engages a [2Fe-2S] cluster (Fig. 3C). This cluster does not appear to have a catalytic role, but rather stabilizes FECH’s tertiary and quaternary structure, enabling the formation of the dimer interface^70^. Indeed, FECH protein is downregulated in several models of Fe-S cluster depletion^71–73^. Whether the Fe-S cluster on FECH has additional functions remains to be explored.

#### Molybdenum cofactor (Moco)

Molybdenum is a vital trace element for animals and is essential for human development. Four key redox enzymes engage in molybdenum-based catalysis in humans: sulfite oxidase, xanthine dehydrogenase, aldehyde oxidase, and mitochondrial amidoxime reductase^74^. However, on its own, molybdenum is biologically inactive, and must be complexed by a scaffold to unlock its biocatalytic potential. The molybdenum cofactor (Moco) is a pterin-based scaffold present in all kingdoms of life, in which molybdenum is coordinated by sulfur. Moco biosynthesis requires the coordinated actions of four human genes (MOCS1, MOCS2, MOCS3, and GEPH sequentially^75^) encoding six proteins that localize to the mitochondria and cytosol. Fe-S clusters play a crucial role in this process^76^, as the mitochondrial MOCS1A enzyme, which coordinates the first step in Moco biosynthesis, binds two [4Fe-4S] clusters per monomer^77^. One cluster has a well-established role in mediating a radical SAM reaction. However, the function of MOCS1A’s auxiliary cluster remains unclear, possibly taking part in substrate coordination. Taken together, Fe-S cluster biosynthesis controls the primary step of Moco production and the activity of Moco-containing enzymes in animals. One exception to this rule was recently discovered in nematodes, when it was demonstrated that *C. elegans* (apart from producing Moco) can import significant amounts of Moco from the bacteria on which it feeds^78,79^. This raises intriguing questions as to whether Moco uptake occurs in other animals, and if any regulatory crosstalk exists between Fe-S clusters depletion and environmental Moco acquisition.

#### Translation

As the respiratory chain is dually encoded by the nuclear and mitochondrial genomes, protein synthesis by matrix-residing mitoribosomes is essential to support mitochondrial bioenergetics^80^. Recent discoveries have uncovered the importance of Fe-S clusters in the mitochondrial translation process (Fig. 3D). Both the mitoribosome assembly factor methyltransferase like protein 17 (METTL17) and the mitoribosomal small subunit (mt-SSU) contain Fe-S clusters. METTL17, a highly conserved protein localized to the mitochondrial matrix^81^, acts as a late-stage assembly factor for the mt-SSU. Biophysical and cryo-EM studies have revealed that METTL17 contains a highly labile [4Fe-4S] cluster- essential for its interaction with the mt-SSU and overall structural stabilization^73,82^. Thus, METTL17 acts as an “Fe-S cluster checkpoint” that matches mitoribosome assembly with Fe-S cluster availability. In addition, structural and functional studies have revealed that the mt-SSU includes two [2Fe-2S] clusters^82–84^. In a rather unique manner, both clusters are simultaneously bound by two different proteins- one engaging with bS18m and bS6m and the other by mS25 and bS16m. Comparative analysis between the mitoribosome and a bacterial homolog highlighted that these 2Fe-2S clusters are found where bacterial ribosomal RNA (rRNA) is typically engaged. This led the authors to the conclusion that these clusters facilitate mt-SSU complex formation, contributing to the stability of newly added proteins in regions where the rRNA has been deleted over the course of evolution.

### Mitochondrial hemoproteins and pathways

#### Steroidogenesis

**Fig. 4:**
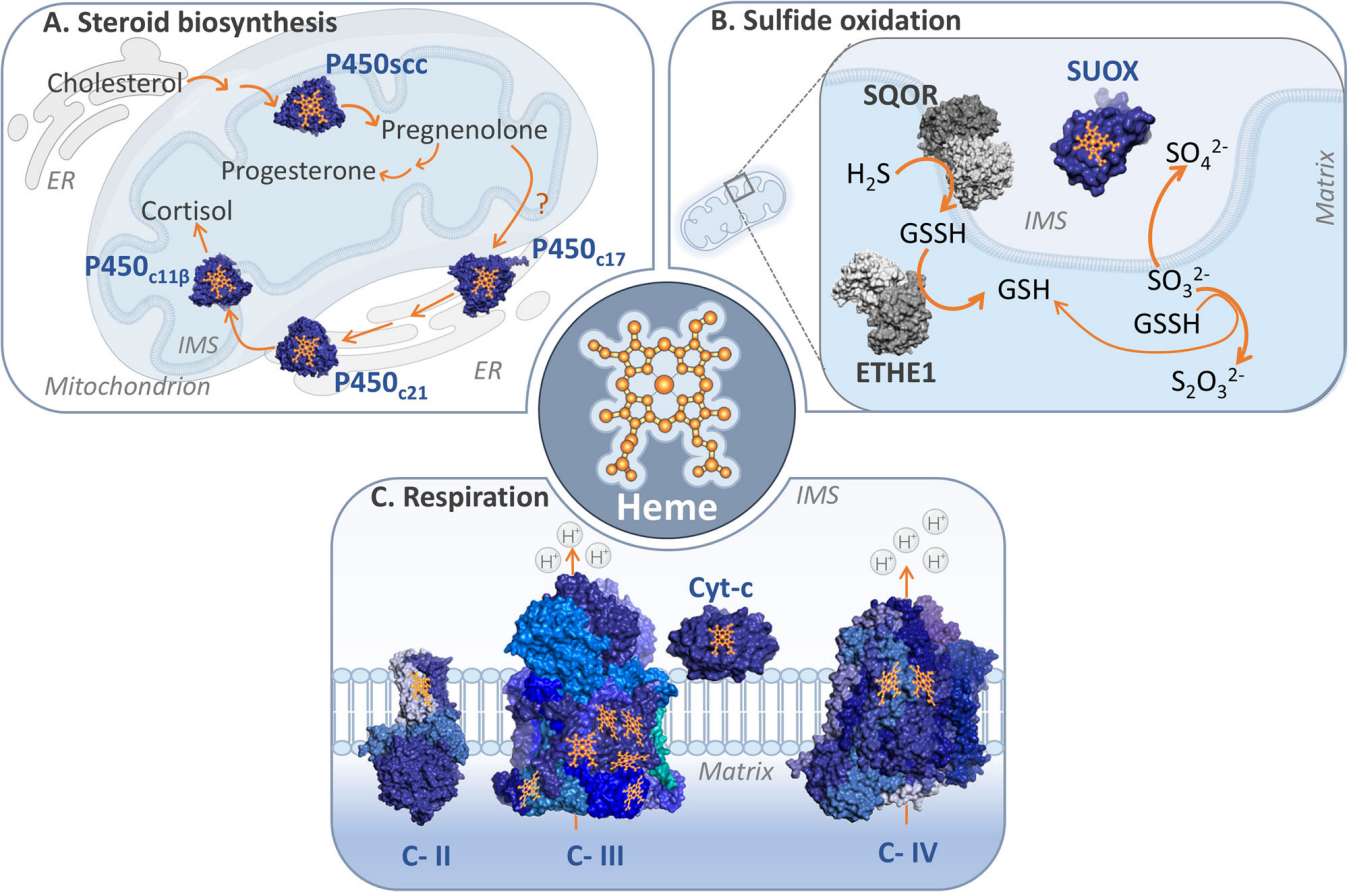
Biological functions of key mitochondrial hemoproteins. **A** Mitochondrial steroid biosynthesis, schematic review depicts the mitochondrial steroidogenesis, involving the transport of cholesterol to the IMM by the StAR protein. Cholesterol is then converted to pregnenolone by the enzyme P450scc through three monooxygenase reactions. Pregnenolone then serves as a precursor for various steroid synthesis, including progesterone and cortisol, with further modifications occurring in the endoplasmic reticulum (ER) and mitochondria. The process relies on the action of the cytochrome P450 family enzymes and the transfer of electrons from NADPH via FDX1; **B** The mitochondrial sulfide oxidation pathway, schematic of the canonical sulfide oxidation pathway in mitochondria, illustrating the reduction of H_2_S to GSSH by SQOR. GSSH is further oxidized by ETHE1 to GSH, to finally produce GSH and S_2_O_3_^2-^. The SO_3_^2-^ metabolite, is alternatively oxidized to SO_4_^2-^ by SUOX; **C** Heme groups’ role of mitochondrial respiration complexes, schematic representation of the electron transfer process within the mitochondrial electron transport chain. Electrons are transferred from Complex II to Complex IV via cytochrome c. The electron shuttles between all enzymes, are transferred to the heme groups fixed at their structure. PDB IDs for protein structures: P450_SCC_(3MZS), P450_C21_(4Y8W), P450_C11β_(6M7X), P450_C17_(6WW0), SUOX(1MJ4), SQOR(6OI5), ETHE1(4CHL), Complex II(8GS8), Complex III(8UGD), Complex IV(5Z62), and Cyt-c(2N9J).

Mitochondria are crucial sites for steroid hormone biosynthesis^85^ (Fig. 4A). Steroidogenesis requires the mobilization of cholesterol to the IMM, where it is subsequently converted into various steroids through a series of reactions involving cytochrome P450 and hydroxysteroid dehydrogenase enzymes^86,87^. Mitochondrial cytochrome P450 enzymes, embedded in the IMM with most of their protein body facing the matrix, bind a single heme moiety^88,89^. By virtue of their redox center, these enzymes function as monooxygenases, activating molecular oxygen using electrons donated by nicotinamide adenine dinucleotide phosphate (NADPH). For example, the first and rate-limiting step in steroidogenesis is the conversion of cholesterol to pregnenolone by a single mitochondrial P450 enzyme, cholesterol side-chain cleavage enzyme (P450scc or CYP11A1)^90^. Thus, P450scc activity serves as a key regulator in the biogenesis of these crucial signaling molecules. Pregnenolone serves as the precursor for multiple different classes of steroid hormones, through divergent reactions that occur in both the mitochondria and ER. Of note, the terminal steps in the biosynthesis of both glucocorticoids and mineralocorticoids occur in the mitochondria. These are mediated by 11β-hydroxylase (P450c11β or CYP11B1), which generates cortisol (followed by activities of the ER enzymes P450c21 and P450c17), and aldosterone synthase (P450c11AS or CYP11B2) which produces aldosterone^91–94^. Cytochrome P450 enzymes cannot oxidize NADPH directly, instead these electrons are relayed via the Fe-S cluster containing protein FDX1^89,95^. In this manner, both iron-based cofactors are essential for steroidogenesis.

#### Hydrogen sulfide oxidation

Hydrogen sulfide (H_2_S) is a two-faced molecule. It can act as a toxin, primarily through its ability to engage heme in Complex IV in the respiratory chain^96^, blocking its activity. Conversely, H_2_S also constitutes a signaling molecule, as revealed by a landmark paper showing its neuromodulatory effects^97^. Thus, its levels must be tightly controlled. The process of H_2_S clearance takes place in the mitochondria (Fig. 4B). The terminal step in this pathway requires a heme- and molybdenum-dependent enzyme named sulfite oxidase (SUOX). In the inner membrane space, SUOX oxidizes sulfite (so_3_^2−^) to sulfate (so_4_^2−^)^98^, which can then be excreted in the urine. Electrons from the H_2_S oxidation contribute to mitochondrial respiration, as SQOR donates its reducing equivalents to coenzyme Q^99^. However, disease-associated SUOX mutations have revealed that heme binding may not be strictly required for SUOX activity^100^. Moreover, H_2_S can also be oxidized by additional proteins, among them heme-containing cytochrome c^101^. This alternate mode of clearance may be relevant in cells and tissues with low levels of the H_2_S oxidation pathway. It is thus notable that H_2_S both inhibits and contributes to respiratory chain activity, suggesting that it induces alternative patterns of electron flow in the respiratory chain^102^.

#### The respiratory chain

Heme is crucial for mitochondrial respiration, aiding in both electron transfer and structural stability in complexes II, III, and IV (Fig. 4C).

One heme moiety is situated between the two transmembrane proteins of complex II, specifically SDHC and SDHD. Initially, it was hypothesized that this heme, along with the Fe-S clusters, would be part of the redox chain that mediates electron transfer from succinate to CoQ. However, subsequent structural studies revealed that the distances between the redox centers make it unfavorable for heme to be involved in electron transfer^103^. Therefore, this heme is now considered to be primarily structural^104^, although it has been speculated that it may play additional regulatory roles.

Complex III contains several heme moieties that form two alternate redox chains, generating the bifurcating Q cycle^105^. In this manner, the oxidation of one CoQH_2_ molecule is coupled with the reduction of cytochrome c and CoQ at opposing sides of the complex. The high-potential redox chain, discussed in section “The respiratory chain”, is made up of one heme on cytochrome *c*_1_ and the Fe-S cluster engaged by UQCRFS1. As previously mentioned, this chain passes the first electron from CoQH_2_ to cytochrome c. The low-potential redox chain is composed of two hemes bound to cytochrome *b*. It transfers the second electron from the CoQH^•^ anion to another CoQ^106,107^. The cycle then repeats. How is this bifurcation enabled, or in other words, why don’t both electrons follow the same path? It’s been shown that once the Fe-S cluster on UQCRFS1 is reduced, this protein undergoes a dramatic conformational change^108–110^. This distances the [2Fe-2S] cluster from the CoQH^•^ anion, making electron transfer more favorable through the low-potential redox chain.

Cytochrome c, a soluble protein localized on the IMM, contains a single heme group. By shuttling electrons one at a time from complexes III to IV, it serves as a crucial linker in the respiratory chain^111^. The heme moiety in cytochrome c is covalently attached to the protein via a conserved CXXCH motif by the enzyme holocytochrome c synthase (HCCS). It is thought that such covalent linkages provide stability, making cytochrome c less likely to lose its cofactor^112^. In the mature protein, one edge of the heme group is positioned ~5 Ȧ from the surface of the protein^113,114^, resulting in small edge-to-edge distances with the redox centers of complex III and IV.

Upon reaching complex IV (cytochrome c oxidase), cytochrome c transfers its electron to a bi-copper center located in the mitochondrially encoded MT-COX2 subunit^115^. This electron is subsequently channeled through three redox centers in MT-COX1. This core subunit is membrane-bound and contributes to both proton pumping and oxygen reduction reactions. To this end, MT-COX1 binds two heme groups and an additional copper ion. A binuclear redox center, made of one heme and copper found ~5 Ȧ apart^116–118^, is the site of molecular oxygen binding and reduction to water. This process is coupled to the flow of four protons across the IMM^119^ through a hydrated channel in MT-COX1^120^. In this manner, heme molecules are central for the terminal step of the respiratory chain, metabolizing ~90% of molecular oxygen found within the cell.

### Mitochondrial iron-binding proteins

Notably, a handful of mitochondrial proteins engage iron directly.

Mitochondrial 5-demethoxyubiquinone hydroxylase (COQ7), a soluble protein found on the matrix side of the IMM, is a conserved enzyme essential for ubiquinone biosynthesis. Part of a multiprotein CoQ metabolon^121^, COQ7 bears a di-iron center that catalyzes penultimate step of ubiquinone biosynthesis^122–125^. Biochemical analysis has revealed that NADH can directly serve as a reductant for COQ7, with no requirement for an additional reductase protein component.

The ethylmalonic encephalopathy 1 protein (ETHE1) is an iron-coordinating metalloprotein that takes part in sulfide catabolism^126,127^. Localized in the mitochondrial matrix, ETHE1 oxidizes glutathione persulfide to sulfite (SO_3_^2−^) while consuming molecular oxygen and water^128^. The mononuclear iron in ETHE1 likely takes part in catalysis, being found at the active site and near the product^127,129^.

## Signaling mitochondrial iron depletion

Attenuated mitochondrial iron stores trigger several cellular homeostasis signaling cascades. Collectively, these counterbalancing measures act to elevate iron availability, while simultaneously blunting global protein translation and activating a distinct transcriptional stress response program. In this manner, such signaling responses enable cells to survive acute phases of iron depletion.

### Iron starvation response and ferritinophagy

Cells have evolved sophisticated sensing pathways to detect and rectify iron deficiencies^130^. The absence of this element may arise from either a scarcity of environmental iron or a failure in uptake, as in the case of lysosomal dysfunction^131^. Changes in its typically acidic pH leads to iron retention within the lysosome, resulting in impaired mitochondrial function^132^.

In metazoan cells, iron homeostatic mechanisms include post-transcriptional response pathways and targeted release of intracellular iron stores. Intriguingly, both regulatory pathways are not responsive to iron directly, but instead react to Fe-S cluster levels^133^.

First, post-transcriptional control of iron levels is carried out by the iron-regulatory proteins, IRP1 and 2^134^. Together, these orthologous proteins regulate the translation and stability of mRNAs bearing an iron response element. This mRNA motif is found in the untranslated regions of key iron metabolism transcripts, capable of modulating cellular iron uptake and storage^135^. How are IRP1/2 activated during iron deficiency? IRP1 binds a [4Fe-4S] cluster at its mRNA binding pocket, so that it can only engage the iron-responsive elements in the absence of this cofactor^136^. Conversely, IRP2 is degraded by a [2Fe-2S] cluster-binding E3 ubiquitin ligase^137^, so that its protein levels are significantly elevated during iron scarcity. IRP2 has also been shown to sense Fe-S cluster levels regardless of its degradation^138^, through a yet-unclear mechanism.

Second, excess intracellular iron is sequestered by cytosolic ferritin, and can be dynamically released upon iron depletion via ‘ferritinophagy’^139,140^. This selective autophagic pathway targets ferritin to the lysosome through the actions of the nuclear receptor coactivator 4 (NCOA4)^141,142^. It was recently discovered that NCOA4 engages a [3Fe-4S] cluster^143,144^. In the absence of its Fe-S cluster, NCOA4 is stabilized and can engage with ferritin. These two mechanisms are in line with the iron accumulation observed in various tissue samples of Fe-S cluster deficiency patients. In this manner, Fe-S clusters serve as pivotal gauges of cellular iron stores.

### Integrated stress response

Studies in various models of mitochondrial dysfunction have consistently identified an activation of the integrated stress response (ISR). This pathway involves the phosphorylation of eukaryotic translation initiation factor 2 alpha (eIF2α) by one of the four eIF2α kinases, reducing global protein synthesis and activating transcription factor ATF4 to restore homeostasis^145,146^. Each of these four kinases is triggered by a specific insult^147^, such as amino acid deficiency^148,149^, unfolded protein stress^150^, heme depletion^151^, and viral infection^152,153^.

Until recently, it was not clear how mitochondrial dysfunction triggers the ISR. There was evidence that the secondary depletion of amino acids caused by blocked respiration might be the primary cause of ISR activation^154^. However, this phenomenon was observed in some contexts, but not others, hinting that alternate ISR activation pathways may exist. Breakthrough work in 2020 showed that it is, in fact, heme-regulated eIF2α kinase (HRI) that is responsive to mitochondrial stress^155,156^. Classically, HRI has been studied in the context of heme depletion. However, under mitochondrial stress, HRI activation occurs through a distinct mechanism, which centers on the mitochondrial “signal relay” protein DELE1. Mitochondrial stress stimulates the cleavage of DELE1 by the protease OMA1, resulting in the accumulation of the C-terminal domain of DELE1 in the cytosol. This short form of DELE1 then binds and activates HRI, initiating the long-observed ISR transcriptional response.

A recent study by the Sekine group demonstrated that under iron deficiency, the HRI-ISR cascade is also activated^157^. Surprisingly, this is not due to heme-induced activation of HRI, but rather triggered by DELE1, although there are some key differences with the established model. In this context, DELE1 is not cleaved by OMA1, but rather is stabilized on mitochondria due to mitochondrial import arrest^157^. This stabilization results in the exposure of DELE1’s C-terminal domain to the cytosol, where it interacts with and activates the HRI-ISR pathway. The Chan group have further extended these findings to show that HRI (and phospho- eIF2α) recruitment to the mitochondrial outer surface also triggers autophagy^158^. Taken together, it appears that mitochondria have co-opted the ISR as a local iron monitoring system, which can mark and eliminate organelles critically depleted for this essential metal.

OMA1-DELE1-dependent ISR signaling may also protect against ferroptosis^159^, an iron-dependent form of cell death^160^ that is further discussed in the section “Mitochondrial iron at the crossroads with human diseases”. Recent work has demonstrated that early-onset cardiomyopathy caused by respiratory chain dysfunction activates the ISR signaling cascade. This response acts to delay pathology and suppresses ferroptosis, by promoting antioxidative factors, such as glutathione^159^. This raises intriguing questions as to the protective role of the ISR in other contexts of iron overload.

## Mitochondrial iron at the crossroads with human diseases

Given the diverse and vital roles of mitochondrial iron, it is not surprising that imbalances can have pathological consequences. These span the range from severe but rare genetically encoded disorders to more common diseases^161,162^. Here, we highlight human pathologies that result from genetic or environmental alterations in mitochondrial iron handling pathways.

### Fe-S cluster deficiency

Mutations in genes related to Fe-S cluster biogenesis or transfer can cause various pathologies. The most prominent Fe-S cluster disorder is Friedreich’s ataxia^163^, which affects 1:50,000 individuals. This autosomal recessive disease is caused by mutations in the nuclear gene FXN^164,165^, which encodes for an allosteric activator of Fe-S cluster biosynthesis in the mitochondria. While the hallmark of this disease is ataxia, patients are also prone to diabetes, scoliosis, hearing and vision loss, and cardiomyopathy^166–169^. The latter is the leading cause of premature mortality in Friedreich’s ataxia^170^. In 2023, Omaveloxolone received FDA clearance for Friedreich’s ataxia^171^, making it the first drug approved for any monogenic mitochondrial disease^172^. Omavaloxolone engages the cell’s endogenous antioxidant response, and while it can attenuate the progression of Friedreich’s ataxia, it is not curative. Thus, additional treatment modalities are actively being investigated for this disease. In this context, chronic hypoxia treatment has shown some promise in various preclinical models of Friedreich’s ataxia, indicating a key role for oxygen in the pathogenesis^173,174^.

Fe-S transfer diseases include X-linked sideroblastic anemia with ataxia (XLSA/A), caused by a mutation in the ABCB7 transporter. The term ‘sideroblastic’ denotes the accumulation of iron-rich mitochondria ringing the nucleus in affected cells- ergo, mitochondria contain iron, but it is not utilized. XLSA/A is characterized by symptoms such as ataxia and cerebellar atrophy hypoplasia^161,175,176^, which usually present within the first 2 years of life. Six distinct mutations in ABCB7 have been reported to cause XLSA/A^177–180^. A deeper understanding of the substrate and transport mechanisms of ABCB7 is crucial to gain insight into the disease’s molecular underpinnings^181,182^.

### Heme depletion

Heme, essential for oxygen transport, cellular respiration, and gene regulation, is mainly produced by erythroblasts and hepatocytes. Heme depletion can either be caused by environmental or genetic disruptions. Iron deficiency-induced anemia is estimated to affect one-third of the global population^183^. In the absence of iron, and subsequent depletion of heme, the HRI kinase is activated in red blood cells^184,185^. HRI acts to block global translation, while activating the ISR. Conversely, mutations in each of the eight heme biosynthetic enzymes cause different systemic implications^30,186^. The most common disorder is X-linked sideroblastic anemia (XLSA), caused by mutations in 5-aminolevulinic acid synthetase 2 (ALAS2), the initial rate-limiting step in erythroid heme synthesis. XLSA occurs mostly in males, with some cases in heterozygous female carriers. While pyridoxine, a cofactor for ALAS2, is a common treatment, not all patients respond to it. Recent studies have nominated DNA demethylating agents as a novel therapeutic approach^187^. Other enzyme defects in the heme biosynthesis pathway are associated with porphyria, classified as either erythropoietic or hepatic based on the site of intermediate product accumulation^188^.

### Neurodegenerative diseases

Pathological iron accumulation is associated with several neurodegenerative diseases, including Alzheimer’s and Parkinson’s diseases. While these elevated iron levels are likely not the primary cause of these disorders, they may contribute to neuronal death. Moreover, the contributions of mitochondrial iron imbalances to neurodegeneration in these different disease contexts remains largely unexplored.

#### Alzheimer’s disease

Alzheimer’s disease (AD) is the most common form of dementia worldwide, estimated to account for 60–80% of cases^189^. AD is characterized by neuron degeneration in various cortical regions, leading to progressive memory loss and dementia. Indeed, changes in brain iron levels can already be detected in the early preclinical stages of AD^190^. In line with this finding, different measures of brain iron load have been shown to be prognostic biomarkers for predicted future cognitive decline and brain atrophy in AD^191–193^. Increased iron load in AD patients has been proposed to contribute to oxidative stress, both within neurons and glia, as well as amyloid plaque aggregation. Another AD marker, TAU, aggregates through heme oxygenase 1 overexpression and iron accumulation^194^. Dysregulated energy metabolism in the brain, driven by insulin resistance, is another key factor in AD progression and is linked to iron accumulation. This metabolic disruption contributes to the formation of amyloid plaques and tau pathology^195,196^. Given the role of iron accumulation in AD, iron chelators have been proposed as a potential therapeutic approach, with evidence suggesting they may inhibit amyloid aggregation^197,198^. Despite these indications, a randomized clinical trial investigating the effects of iron chelation on AD progression found that reducing iron levels accelerated cognitive decline and increased regional brain atrophy^199^. This surprising result raises the possibility that iron bioavailability is, in fact, limiting in AD.

#### Parkinson’s disease

Parkinson’s disease (PD), the most common neurodegenerative movement disorder, is characterized by accelerated neuronal death of primarily dopaminergic neurons and the accumulation of α-synuclein^200^. Already in 1923, German pathologist Friedrich Lewy described perivascular iron deposits in postmortem PD brains^201^. These findings have since been repeated many times over^202–205^. It has been proposed that PD iron overload can act as a driver of oxidative stress and iron-mediated cell death, i.e. ‘ferroptosis’. Moreover, iron and α-synuclein^200^ are connected both at the translational and protein level. First, the α-synuclein transcript was reported to contain an iron response element at its 5’UTR^206^, elevating its translation upon iron excess. Second, ferric iron can trigger α-synuclein aggregation^207^, and α-synuclein aggregates analyzed from postmortem brain samples are iron-laden^208^. However, in 2022, a PD clinical trial with the iron chelator deferiprone showed that, despite effectively reducing brain iron levels, deferiprone was associated with worsening motor and nonmotor symptoms^209^. In sum, while a link between iron accumulation and PD exists, the pathophysiological mechanisms of iron deposition and its effects on disease progression require additional study.

### Mitochondrial iron and ferroptosis

Ferroptosis is an iron-dependent form of cell death, in which iron reacts with oxygen to drive the accumulation of lipid peroxides^160^. The oxidized plasma membrane becomes permeable to cations, culminating in cell swelling and plasma membrane rupture^210–212^. Multiple pathways can counteract the toxic accumulation of lipid peroxides, chief among them being the enzyme glutathione peroxidase 4 (GPX4). GPX4 can convert lipid hydroperoxides to less dangerous lipid alcohols, a reaction that depends on GSH^213,214^. Additional ferroptosis buffering pathways include ferroptosis suppressor protein 1 (FSP1), which regenerates reducing elements in the plasma membrane, such as CoQ or vitamin K, that can terminate the lipid peroxidation process. However, these ferroptotic-defense mechanisms can be overwhelmed, likely contributing to both rare and common pathologies, such as sedaghatian-type spondylometaphyseal dysplasia^215^, age-related macular degeneration^216^, and hereditary hemochromatosis^217^.

Given their central role in cellular iron and redox metabolism, it is not surprising that mitochondria are potent modulators of ferroptosis^218^. Mitochondria mediate CoQ biosynthesis and the breakdown of polyunsaturated fatty acids, which are the main source of possible lipid peroxides. CISD1, a 2Fe-2S cluster protein located in the mitochondrial outer membrane, protects against mitochondrial lipid peroxidation and thereby inhibits ferroptosis^219^. However, the mitochondria can also potentiate ferroptosis under some conditions. Upon cysteine deprivation, mitochondrial metabolism contributes to rapid glutathione depletion, leading to cellular lipid peroxide accumulation and subsequent ferroptosis^220^. In addition, disruptions in mitochondrial Fe-S cluster biosynthesis lead to the activation of the iron starvation response, elevating intracellular iron levels and the susceptibility to ferroptosis^221,222^. Taken together, the contribution of mitochondrial iron handling to ferroptosis is complex and likely context-dependent.

## Future outlook

The past 25 years of research have revolutionized our understanding of the ways in which mitochondria metabolize iron, and how this metal, in turn, unlocks manifold abilities for the mitochondria. Future research will have to tackle important questions that remain unsolved: What is the entire spectrum of mitochondrial iron metalloproteins, whether they bind to Fe-S clusters, heme, or iron ions directly? These proteins are still being identified on an ad-hoc basis, and so we lack a comprehensive view of how iron sustains and regulates this ancient organelle. How is iron partitioned between the various pathways that it can support in the mitochondria? Does mitochondrial iron depletion induce additional signaling or restorative pathways, either within the mitochondria or extending into the cytosol? Can mitochondria communicate iron status directly to other organelles, e.g., the lysosome which functions as a key iron entry point? Can iron be exported from mitochondria? Other than in the context of heme, iron export pathways remain ill-defined. Why do certain tissues exhibit heightened vulnerability to mitochondrial iron dyshomeostasis? This variability likely stems from tissue-specific differences in iron demand, metabolic activity, regulation, and susceptibility to iron-induced cellular damage that requires further clarification. Deepening this understanding will provide an important framework for the development of therapeutic modalities that counteract iron-linked diseases.

## Data Availability

No datasets were generated or analysed during the current study.

## References

[1] Anbar, A. D. Oceans. Elements and evolution. *Science* **322**, 1481–1483 (2008). 19056967 10.1126/science.1163100

[2] Goldford, J. E. et al. Remnants of an ancient metabolism without phosphate. *Cell* **168**, 1126–1134.e9 (2017). 28262353 10.1016/j.cell.2017.02.001

[3] Muchowska, K. B., Varma, S. J. & Moran, J. Synthesis and breakdown of universal metabolic precursors promoted by iron. *Nature* **569**, 104–107 (2019). 31043728 10.1038/s41586-019-1151-1 PMC6517266

[4] Andreini, C. et al. The human iron-proteome. *Metallomics* **10**, 1223–1231 (2018). 30095136 10.1039/c8mt00146d

[5] Liu, J. et al. Metalloproteins containing cytochrome, iron-sulfur, or copper redox centers. *Chem. Rev.* **114**, 4366–4469 (2014). 24758379 10.1021/cr400479b PMC4002152

[6] Lill, R., et al. Is there an answer? Why are mitochondria essential for life? *IUBMB Life* **57**, 701–703 (2005). 10.1080/15216540500305860 16223711

[7] Galy, B., Conrad, M. & Muckenthaler, M. Mechanisms controlling cellular and systemic iron homeostasis. *Nat. Rev. Mol. Cell Biol.*10.1038/s41580-023-00648-1 (2023). 10.1038/s41580-023-00648-1 37783783

[8] Ward, D. M. & Cloonan, S. M. Mitochondrial Iron in Human Health and Disease. *Annu. Rev. Physiol.* **81**, 453–482 (2019). 30485761 10.1146/annurev-physiol-020518-114742 PMC6641538

[9] Beinert, H., Holm, R. H. & Munck, E. Iron-sulfur clusters: nature’s modular, multipurpose structures. *Science* **277**, 653–659 (1997). 9235882 10.1126/science.277.5326.653

[10] Ichiye, T. In *Iron-Sulfur Clusters in Chemistry and Biology* (ed. Tracey, R.) Ch. 2 (De Gruyter, 2014).

[11] Bonomi, F. & Rouault, T. A. In *Iron-Sulfur Clusters in Chemistry and Biology* (ed. Tracey, R.) Ch. 1 (De Gruyter: Berlin, 2014).

[12] Kennedy, M. C. et al. The role of iron in the activation-inactivation of aconitase. *J. Biol. Chem.* **258**, 11098–11105 (1983). 6309829

[13] Venkateswara Rao, P. & Holm, R. H. Synthetic analogues of the active sites of iron-sulfur proteins. *Chem. Rev.* **104**, 527–559 (2004). 14871134 10.1021/cr020615+

[14] Zheng, L. et al. Cysteine desulfurase activity indicates a role for NIFS in metallocluster biosynthesis. *Proc. Natl Acad. Sci. USA* **90**, 2754–2758 (1993). 8464885 10.1073/pnas.90.7.2754 PMC46174

[15] Zheng, L. et al. Assembly of iron-sulfur clusters. Identification of an iscSUA-hscBA-fdx gene cluster from Azotobacter vinelandii. *J. Biol. Chem.* **273**, 13264–13272 (1998). 9582371 10.1074/jbc.273.21.13264

[16] Kispal, G. et al. The mitochondrial proteins Atm1p and Nfs1p are essential for biogenesis of cytosolic Fe/S proteins. *EMBO J.* **18**, 3981–3989 (1999). 10406803 10.1093/emboj/18.14.3981 PMC1171474

[17] Maio, N. & Rouault, T. A. Mammalian Fe-S proteins: definition of a consensus motif recognized by the co-chaperone HSC20. *Metallomics* **8**, 1032–1046 (2016). 27714045 10.1039/c6mt00167j PMC5240853

[18] Naamati, A. et al. Dual targeting of Nfs1 and discovery of its novel processing enzyme, Icp55. *J. Biol. Chem.* **284**, 30200–30208 (2009). 19720832 10.1074/jbc.M109.034694 PMC2781575

[19] Kim, K. S. et al. Cytosolic HSC20 integrates de novo iron-sulfur cluster biogenesis with the CIAO1-mediated transfer to recipients. *Hum. Mol. Genet* **27**, 837–852 (2018). 29309586 10.1093/hmg/ddy004 PMC6075588

[20] Tong, W. H. & Rouault, T. A. Functions of mitochondrial ISCU and cytosolic ISCU in mammalian iron-sulfur cluster biogenesis and iron homeostasis. *Cell Metab.* **3**, 199–210 (2006). 16517407 10.1016/j.cmet.2006.02.003

[21] Weiler, B. D. et al. Mitochondrial [4Fe-4S] protein assembly involves reductive [2Fe-2S] cluster fusion on ISCA1-ISCA2 by electron flow from ferredoxin FDX2. *Proc. Natl Acad. Sci. USA* **117**, 20555–20565 (2020). 10.1073/pnas.2003982117 PMC7456137 32817474

[22] Swenson, S. A. et al. From synthesis to utilization: the ins and outs of mitochondrial heme. *Cells* **9**, 579 (2020). 10.3390/cells9030579 PMC7140478 32121449

[23] Immenschuh, S. et al. Heme as a target for therapeutic interventions. *Front. Pharm.* **8**, 146 (2017). 10.3389/fphar.2017.00146 PMC5378770 28420988

[24] Gebicka, L. Redox reactions of heme proteins with flavonoids. *J. Inorg. Biochem.* **208**, 111095 (2020). 32442763 10.1016/j.jinorgbio.2020.111095

[25] Rao, A. U. et al. Lack of heme synthesis in a free-living eukaryote. *Proc. Natl Acad. Sci. USA* 102, 4270–4275 (2005). 10.1073/pnas.0500877102 PMC555530 15767563

[26] Stojanovski, B. M. et al. 5-Aminolevulinate synthase catalysis: the catcher in heme biosynthesis. *Mol. Genet. Metab.* **128**, 178–189 (2019). 31345668 10.1016/j.ymgme.2019.06.003 PMC6908770

[27] Bayeva, M. et al. ATP-binding cassette B10 regulates early steps of heme synthesis. *Circ. Res.* **113**, 279–287 (2013). 23720443 10.1161/CIRCRESAHA.113.301552 PMC3817742

[28] Hewton, K. G., Johal, A. S. & Parker, S. J. Transporters at the interface between cytosolic and mitochondrial amino acid metabolism. *Metabolites* **11**, 112 (2021). 10.3390/metabo11020112 PMC7920303 33669382

[29] Yien, Y. Y. & Perfetto, M. Regulation of heme synthesis by mitochondrial homeostasis proteins. *Front. Cell Dev. Biol.* **10**, 895521 (2022). 35832791 10.3389/fcell.2022.895521 PMC9272004

[30] Fujiwara, T. & Harigae, H. Biology of heme in mammalian erythroid cells and related disorders. *Biomed. Res. Int.* **2015**, 278536 (**2015**). p. 26557657 10.1155/2015/278536 PMC4628764

[31] Obi, C. D. et al. Ferrochelatase: mapping the intersection of iron and porphyrin metabolism in the mitochondria. *Front. Cell Dev. Biol.* **10**, 894591 (2022). 35646904 10.3389/fcell.2022.894591 PMC9133952

[32] Jhurry, N. D. et al. Biophysical investigation of the ironome of human jurkat cells and mitochondria. *Biochemistry* **51**, 5276–5284 (2012). 22726227 10.1021/bi300382d PMC3448029

[33] Rauen, U. et al. Assessment of chelatable mitochondrial iron by using mitochondrion-selective fluorescent iron indicators with different iron-binding affinities. *Chembiochem* **8**, 341–352 (2007). 17219451 10.1002/cbic.200600311

[34] Levi, S. et al. Mitochondrial ferritin: its role in physiological and pathological conditions. *Cells* **10**, 1969 (2021). 10.3390/cells10081969 PMC8393899 34440737

[35] Finazzi, D. & Arosio, P. Biology of ferritin in mammals: an update on iron storage, oxidative damage and neurodegeneration. *Arch. Toxicol.* **88**, 1787–1802 (2014). 25119494 10.1007/s00204-014-1329-0

[36] Bartnikas, T. B. et al. Characterization of mitochondrial ferritin-deficient mice. *Am. J. Hematol.* **85**, 958–960 (2010). 20960432 10.1002/ajh.21872 PMC3319725

[37] Wang, P. et al. Mitochondrial ferritin attenuates cerebral ischaemia/reperfusion injury by inhibiting ferroptosis. *Cell Death Dis.* **12**, 447 (2021). 33953171 10.1038/s41419-021-03725-5 PMC8099895

[38] Wang, P. et al. Mitochondrial ferritin deletion exacerbates beta-amyloid-induced neurotoxicity in mice. *Oxid. Med. Cell Longev.* **2017**, 1020357 (**2017**). 28191272 10.1155/2017/1020357 PMC5278219

[39] Wang, P. et al. Mitochondrial ferritin alleviates apoptosis by enhancing mitochondrial bioenergetics and stimulating glucose metabolism in cerebral ischemia reperfusion. *Redox Biol.* **57**, 102475 (2022). 36179435 10.1016/j.redox.2022.102475 PMC9526171

[40] Shi, Z. H. et al. Neuroprotective mechanism of mitochondrial ferritin on 6-hydroxydopamine-induced dopaminergic cell damage: implication for neuroprotection in Parkinson’s disease. *Antioxid. Redox Signal* **13**, 783–796 (2010). 20121342 10.1089/ars.2009.3018 PMC6777976

[41] Santambrogio, P. et al. Mitochondrial ferritin expression in adult mouse tissues. *J. Histochem. Cytochem.* **55**, 1129–1137 (2007). 17625226 10.1369/jhc.7A7273.2007 PMC3957534

[42] Yanatori, I. et al. Newly uncovered biochemical and functional aspects of ferritin. *FASEB J.* **37**, e23095 (2023). 37440196 10.1096/fj.202300918R

[43] Plays, M., Muller, S. & Rodriguez, R. Chemistry and biology of ferritin. *Metallomics* **13**, mfab021 (2021) . 10.1093/mtomcs/mfab021 PMC8083198 33881539

[44] Vercellino, I. & Sazanov, L. A. The assembly, regulation and function of the mitochondrial respiratory chain. *Nat. Rev. Mol. Cell Biol.* **23**, 141–161 (2022). 34621061 10.1038/s41580-021-00415-0

[45] Braymer, J. J. & Lill, R. Iron-sulfur cluster biogenesis and trafficking in mitochondria. *J. Biol. Chem.* **292**, 12754–12763 (2017). 28615445 10.1074/jbc.R117.787101 PMC5546016

[46] Scheffler, I. E. Mitochondrial disease associated with complex I (NADH-CoQ oxidoreductase) deficiency. *J. Inherit. Metab. Dis.* **38**, 405–415 (2015). 25224827 10.1007/s10545-014-9768-6

[47] Gnandt, E. et al. The multitude of iron-sulfur clusters in respiratory complex I. *Biochim. Biophys. Acta* **1857**, 1068–1072 (2016). 26944855 10.1016/j.bbabio.2016.02.018

[48] Fiedorczuk, K. et al. Atomic structure of the entire mammalian mitochondrial complex I. *Nature* **538**, 406–410 (2016). 27595392 10.1038/nature19794 PMC5164932

[49] Zhu, J., Vinothkumar, K. R. & Hirst, J. Structure of mammalian respiratory complex I. *Nature* **536**, 354–358 (2016). 27509854 10.1038/nature19095 PMC5027920

[50] Hinton, T. V. et al. Molecular characteristics of proteins within the mitochondrial Fe-S cluster assembly complex. *Micron* **153**, 103181 (2022). 34823116 10.1016/j.micron.2021.103181

[51] Mosegaard, S. et al. An intronic variation in SLC52A1 causes exon skipping and transient riboflavin-responsive multiple acyl-CoA dehydrogenation deficiency. *Mol. Genet. Metab.* **122**, 182–188 (2017). 29122468 10.1016/j.ymgme.2017.10.014

[52] Baik, A. H. et al. Oxygen toxicity causes cyclic damage by destabilizing specific Fe-S cluster-containing protein complexes. *Mol. Cell* **83**, 942–960 e9 (2023). 36893757 10.1016/j.molcel.2023.02.013 PMC10148707

[53] Bergner, M. et al. Model of the MitoNEET [2Fe-2S] cluster shows proton coupled electron transfer. *J. Am. Chem. Soc*. 139, 701–707 (2017). 10.1021/jacs.6b09180 PMC5812485 28055193

[54] Wang, Y., Landry, A. P. & Ding, H. The mitochondrial outer membrane protein mitoNEET is a redox enzyme catalyzing electron transfer from FMNH(2) to oxygen or ubiquinone. *J. Biol. Chem.* **292**, 10061–10067 (2017). 28461337 10.1074/jbc.M117.789800 PMC5473213

[55] Nechushtai, R. et al. CISD3/MiNT is required for complex I function, mitochondrial integrity, and skeletal muscle maintenance. *Proc. Natl Acad. Sci. USA* **121**, e2405123121 (2024). 38781208 10.1073/pnas.2405123121 PMC11145280

[56] Kang, W. et al. Emerging role of TCA cycle-related enzymes in human diseases. *Int. J. Mol. Sci*. **22**, 13057 (2021). 10.3390/ijms222313057 PMC8657694 34884868

[57] Maio, N., Heffner, A. L. & Rouault, T. A. Iron‑sulfur clusters in viral proteins: exploring their elusive nature, roles and new avenues for targeting infections. *Biochim. Biophys. Acta Mol. Cell Res.* **1871**, 119723 (2024). 38599324 10.1016/j.bbamcr.2024.119723 PMC11139609

[58] McCarthy, E. L. & Booker, S. J. Destruction and reformation of an iron-sulfur cluster during catalysis by lipoyl synthase. *Science* **358**, 373–377 (2017). 29051382 10.1126/science.aan4574 PMC5941298

[59] Dreishpoon, M. B. et al. FDX1 regulates cellular protein lipoylation through direct binding to LIAS. *J. Biol. Chem.* **299**, 105046 (2023). 37453661 10.1016/j.jbc.2023.105046 PMC10462841

[60] Joshi, P. R. et al. Lipoylation is dependent on the ferredoxin FDX1 and dispensable under hypoxia in human cells. *J. Biol. Chem.* **299**, 105075 (2023). 37481209 10.1016/j.jbc.2023.105075 PMC10470009

[61] Schulz, V. et al. Functional spectrum and specificity of mitochondrial ferredoxins FDX1 and FDX2. *Nat. Chem. Biol.* **19**, 206–217 (2023). 36280795 10.1038/s41589-022-01159-4 PMC10873809

[62] Murphy, M. P. How mitochondria produce reactive oxygen species. *Biochem. J.* **417**, 1–13 (2009). 19061483 10.1042/BJ20081386 PMC2605959

[63] Griffith, O. W. & Meister, A. Origin and turnover of mitochondrial glutathione. *Proc. Natl Acad. Sci. USA* **82**, 4668–4672 (1985). 3860816 10.1073/pnas.82.14.4668 PMC390447

[64] Wang, Y. et al. SLC25A39 is necessary for mitochondrial glutathione import in mammalian cells. *Nature* **599**, 136–140 (2021). 34707288 10.1038/s41586-021-04025-w PMC10981497

[65] Shi, X. et al. Combinatorial GxGxE CRISPR screen identifies SLC25A39 in mitochondrial glutathione transport linking iron homeostasis to OXPHOS. *Nat. Commun.* **13**, 2483 (2022). 35513392 10.1038/s41467-022-30126-9 PMC9072411

[66] Liu, Y. et al. Autoregulatory control of mitochondrial glutathione homeostasis. *Science* **382**, 820–828 (2023). 37917749 10.1126/science.adf4154 PMC11170550

[67] Shi, X. et al. Dual regulation of SLC25A39 by AFG3L2 and iron controls mitochondrial glutathione homeostasis. *Mol. Cell* **84**, 802–810 e6 (2024). 38157846 10.1016/j.molcel.2023.12.008 PMC10922821

[68] Liu, G. et al. Heme biosynthesis depends on previously unrecognized acquisition of iron-sulfur cofactors in human amino-levulinic acid dehydratase. *Nat. Commun.* **11**, 6310 (2020). 33298951 10.1038/s41467-020-20145-9 PMC7725820

[69] Dailey, H. A., Finnegan, M. G. & Johnson, M. K. Human ferrochelatase is an iron-sulfur protein. *Biochemistry* **33**, 403–407 (1994). 8286370 10.1021/bi00168a003

[70] Wu, C. K. et al. The 2.0 A structure of human ferrochelatase, the terminal enzyme of heme biosynthesis. *Nat. Struct. Biol.* **8**, 156–160 (2001). 11175906 10.1038/84152

[71] Crooks, D. R. et al. Posttranslational stability of the heme biosynthetic enzyme ferrochelatase is dependent on iron availability and intact iron-sulfur cluster assembly machinery. *Blood* **115**, 860–869 (2010). 19965627 10.1182/blood-2009-09-243105 PMC2815515

[72] Vasquez-Trincado, C. et al. Adaptation of the heart to Frataxin depletion: evidence that integrated stress response can predominate over mTORC1 activation. *Hum. Mol. Genet.* **33**, 637–654 (2021). 34550363 10.1093/hmg/ddab216 PMC11000666

[73] Ast, T. et al. METTL17 is an Fe-S cluster checkpoint for mitochondrial translation. *Mol. Cell* **84**, 359–374.e8 (2024). 38199006 10.1016/j.molcel.2023.12.016 PMC11046306

[74] Atwal, P. S. & Scaglia, F. Molybdenum cofactor deficiency. *Mol. Genet. Metab.* **117**, 1–4 (2016). 26653176 10.1016/j.ymgme.2015.11.010

[75] Schwarz, G. Molybdenum cofactor biosynthesis and deficiency. *Cell Mol. Life Sci.* **62**, 2792–2810 (2005). 16261263 10.1007/s00018-005-5269-y PMC11145942

[76] Hasnat, M. A. & Leimkuhler, S. Shared functions of Fe-S cluster assembly and Moco biosynthesis. *Biochim. Biophys. Acta Mol. Cell Res.* **1871**, 119731 (2024). 38631442 10.1016/j.bbamcr.2024.119731

[77] Hanzelmann, P. et al. Characterization of MOCS1A, an oxygen-sensitive iron-sulfur protein involved in human molybdenum cofactor biosynthesis. *J. Biol. Chem.* **279**, 34721–34732 (2004). 15180982 10.1074/jbc.M313398200

[78] Oliphant, K. D. et al. Obtaining the necessary molybdenum cofactor for sulfite oxidase activity in the nematode *Caenorhabditis elegans* surprisingly involves a dietary source. *J. Biol. Chem.* **299**, 102736 (2023). 36423681 10.1016/j.jbc.2022.102736 PMC9793310

[79] Warnhoff, K. & Ruvkun, G. Molybdenum cofactor transfer from bacteria to nematode mediates sulfite detoxification. *Nat. Chem. Biol.* **15**, 480–488 (2019). 30911177 10.1038/s41589-019-0249-y PMC6470025

[80] Suhm, T. & Ott, M. Mitochondrial translation and cellular stress response. *Cell Tissue Res.* **367**, 21–31 (2017). 27425851 10.1007/s00441-016-2460-4

[81] He, J. et al. Human C4orf14 interacts with the mitochondrial nucleoid and is involved in the biogenesis of the small mitochondrial ribosomal subunit. *Nucleic Acids Res.* **40**, 6097–6108 (2012). 22447445 10.1093/nar/gks257 PMC3401442

[82] Zhong, H. et al. BOLA3 and NFU1 link mitoribosome iron-sulfur cluster assembly to multiple mitochondrial dysfunctions syndrome. *Nucleic Acids Res.* **51**, 11797–11812 (2023). 37823603 10.1093/nar/gkad842 PMC10681725

[83] Itoh, Y. et al. Mechanism of mitoribosomal small subunit biogenesis and preinitiation. *Nature* **606**, 603–608 (2022). 35676484 10.1038/s41586-022-04795-x PMC9200640

[84] Itoh, Y. et al. Structure of the mitoribosomal small subunit with streptomycin reveals Fe-S clusters and physiological molecules. *Elife* **11**, e77460 (2022). 10.7554/eLife.77460 PMC9731571 36480258

[85] Bassi, G., Sidhu, S. K. & Mishra, S. The expanding role of mitochondria, autophagy and lipophagy in steroidogenesis. *Cells* **10**, 1851 (2021). 10.3390/cells10081851 PMC8391558 34440620

[86] Midzak, A. & Papadopoulos, V. Adrenal mitochondria and steroidogenesis: from individual proteins to functional protein assemblies. *Front. Endocrinol.* **7**, 106 (2016). 10.3389/fendo.2016.00106 PMC4965458 27524977

[87] Li, J., Papadopoulos, V. & Vihma, V. Steroid biosynthesis in adipose tissue. *Steroids* **103**, 89–104 (2015). 25846979 10.1016/j.steroids.2015.03.016

[88] Zhao, M. et al. Cytochrome P450 enzymes and drug metabolism in humans. *Int. J. Mol. Sci*. **22**, 12808 (2021). 10.3390/ijms222312808 PMC8657965 34884615

[89] Strushkevich, N. et al. Structural basis for pregnenolone biosynthesis by the mitochondrial monooxygenase system. *Proc. Natl Acad. Sci. USA* **108**, 10139–10143 (2011). 21636783 10.1073/pnas.1019441108 PMC3121847

[90] Papadopoulos, V. & Miller, W. L. Role of mitochondria in steroidogenesis. *Best. Pr. Res. Clin. Endocrinol. Metab.* **26**, 771–790 (2012). 10.1016/j.beem.2012.05.002 23168279

[91] Curnow, K. M. et al. The product of the CYP11B2 gene is required for aldosterone biosynthesis in the human adrenal cortex. *Mol. Endocrinol.* **5**, 1513–1522 (1991). 1775135 10.1210/mend-5-10-1513

[92] Strushkevich, N. et al. Structural insights into aldosterone synthase substrate specificity and targeted inhibition. *Mol. Endocrinol.* **27**, 315–324 (2013). 23322723 10.1210/me.2012-1287 PMC5417327

[93] White, P. C. et al. A mutation in CYP11B1 (Arg-448-His) associated with steroid 11 beta-hydroxylase deficiency in Jews of Moroccan origin. *J. Clin. Invest.* **87**, 1664–1667 (1991). 2022736 10.1172/JCI115182 PMC295260

[94] Curnow, K. M. et al. Mutations in the CYP11B1 gene causing congenital adrenal hyperplasia and hypertension cluster in exons 6, 7, and 8. *Proc. Natl Acad. Sci. USA* **90**, 4552–4556 (1993). 8506298 10.1073/pnas.90.10.4552 PMC46550

[95] Sheftel, A. D. et al. Humans possess two mitochondrial ferredoxins, Fdx1 and Fdx2, with distinct roles in steroidogenesis, heme, and Fe/S cluster biosynthesis. *Proc. Natl Acad. Sci. USA* **107**, 11775–11780 (2010). 20547883 10.1073/pnas.1004250107 PMC2900682

[96] Nicholls, P. & Kim, J. K. Sulphide as an inhibitor and electron donor for the cytochrome c oxidase system. *Can. J. Biochem.* **60**, 613–623 (1982). 6288202 10.1139/o82-076

[97] Abe, K. & Kimura, H. The possible role of hydrogen sulfide as an endogenous neuromodulator. *J. Neurosci.* **16**, 1066–1071 (1996). 8558235 10.1523/JNEUROSCI.16-03-01066.1996 PMC6578817

[98] Enemark, J. H. Mechanistic complexities of sulfite oxidase: an enzyme with multiple domains, subunits, and cofactors. *J. Inorg. Biochem.* **247**, 112312 (2023). 37441922 10.1016/j.jinorgbio.2023.112312

[99] Landry, A. P., Ballou, D. P. & Banerjee, R. Hydrogen sulfide oxidation by sulfide quinone oxidoreductase. *Chembiochem* **22**, 949–960 (2021). 33080111 10.1002/cbic.202000661 PMC7969369

[100] Bender, D. et al. Oxygen and nitrite reduction by heme-deficient sulphite oxidase in a patient with mild sulphite oxidase deficiency. *J. Inherit. Metab. Dis.* **43**, 748–757 (2020). 31950508 10.1002/jimd.12216

[101] Vitvitsky, V. et al. Cytochrome c reduction by H(2)S potentiates sulfide signaling. *ACS Chem. Biol.* **13**, 2300–2307 (2018). 29966080 10.1021/acschembio.8b00463 PMC6450078

[102] Kumar, R. et al. A redox cycle with complex II prioritizes sulfide quinone oxidoreductase-dependent H(2)S oxidation. *J. Biol. Chem.* **298**, 101435 (2022). 34808207 10.1016/j.jbc.2021.101435 PMC8683732

[103] Sun, F. et al. Crystal structure of mitochondrial respiratory membrane protein complex II. *Cell* **121**, 1043–1057 (2005). 15989954 10.1016/j.cell.2005.05.025

[104] Oyedotun, K. S., Sit, C. S. & Lemire, B. D. The *Saccharomyces cerevisiae* succinate dehydrogenase does not require heme for ubiquinone reduction. *Biochim. Biophys. Acta* **1767**, 1436–1445 (2007). 18028869 10.1016/j.bbabio.2007.09.008

[105] Mitchell, P. Possible molecular mechanisms of the protonmotive function of cytochrome systems. *J. Theor. Biol.* **62**, 327–367 (1976). 186667 10.1016/0022-5193(76)90124-7

[106] Brzezinski, P., Moe, A. & Adelroth, P. Structure and mechanism of respiratory III-IV supercomplexes in bioenergetic membranes. *Chem. Rev.* **121**, 9644–9673 (2021). 34184881 10.1021/acs.chemrev.1c00140 PMC8361435

[107] Kim, H. J. et al. Structure, function, and assembly of heme centers in mitochondrial respiratory complexes. *Biochim. Biophys. Acta* **1823**, 1604–1616 (2012). 22554985 10.1016/j.bbamcr.2012.04.008 PMC3601904

[108] Muhleip, A. et al. Structural basis of mitochondrial membrane bending by the I-II-III(2)-IV(2) supercomplex. *Nature* **615**, 934–938 (2023). 36949187 10.1038/s41586-023-05817-y PMC10060162

[109] Zhang, Z. et al. Electron transfer by domain movement in cytochrome bc1. *Nature* **392**, 677–684 (1998). 9565029 10.1038/33612

[110] Rajagukguk, S. et al. Effect of mutations in the cytochrome b ef loop on the electron-transfer reactions of the Rieske iron-sulfur protein in the cytochrome bc1 complex. *Biochemistry* **46**, 1791–1798 (2007). 17253777 10.1021/bi062094g PMC2527182

[111] Perez-Mejias, G. et al. Cytochrome c: surfing off of the mitochondrial membrane on the tops of complexes III and IV. *Comput. Struct. Biotechnol. J.* **17**, 654–660 (2019). 31193759 10.1016/j.csbj.2019.05.002 PMC6542325

[112] Kranz, R. G. et al. Cytochrome c biogenesis: mechanisms for covalent modifications and trafficking of heme and for heme-iron redox control. *Microbiol. Mol. Biol. Rev.* **73**, 510–528 (2009). 19721088 10.1128/MMBR.00001-09 PMC2738134

[113] Louie, G. V. & Brayer, G. D. High-resolution refinement of yeast iso-1-cytochrome c and comparisons with other eukaryotic cytochromes c. *J. Mol. Biol.* **214**, 527–555 (1990). 2166169 10.1016/0022-2836(90)90197-T

[114] Alvarez-Paggi, D. et al. Multifunctional cytochrome c: learning new tricks from an old dog. *Chem. Rev.* **117**, 13382–13460 (2017). 29027792 10.1021/acs.chemrev.7b00257

[115] Yoshikawa, S., Muramoto, K. & Shinzawa-Itoh, K. Reaction mechanism of mammalian mitochondrial cytochrome c oxidase. *Adv. Exp. Med. Biol.* **748**, 215–236 (2012). 22729860 10.1007/978-1-4614-3573-0_9

[116] Tsukihara, T. et al. Structures of metal sites of oxidized bovine heart cytochrome c oxidase at 2.8 A. *Science* **269**, 1069–1074 (1995). 7652554 10.1126/science.7652554

[117] Yoshikawa, S. et al. Redox-coupled crystal structural changes in bovine heart cytochrome c oxidase. *Science* **280**, 1723–1729 (1998). 9624044 10.1126/science.280.5370.1723

[118] Tsukihara, T. et al. The whole structure of the 13-subunit oxidized cytochrome c oxidase at 2.8 A. *Science* **272**, 1136–1144 (1996). 8638158 10.1126/science.272.5265.1136

[119] Wikstrom, M. K. Proton pump coupled to cytochrome c oxidase in mitochondria. *Nature* **266**, 271–273 (1977). 15223 10.1038/266271a0

[120] Marechal, A. et al. A common coupling mechanism for A-type heme-copper oxidases from bacteria to mitochondria. *Proc. Natl Acad. Sci. USA* **117**, 9349–9355 (2020). 32291342 10.1073/pnas.2001572117 PMC7196763

[121] Manicki, M. et al. Structure and functionality of a multimeric human COQ7:COQ9 complex. *Mol. Cell* **82**, 4307–4323.e10 (2022). 36306796 10.1016/j.molcel.2022.10.003 PMC10058641

[122] Acosta, M. J. et al. Coenzyme Q biosynthesis in health and disease. *Biochim. Biophys. Acta* **1857**, 1079–1085 (2016). 27060254 10.1016/j.bbabio.2016.03.036

[123] Stenmark, P. et al. A new member of the family of di-iron carboxylate proteins. Coq7 (clk-1), a membrane-bound hydroxylase involved in ubiquinone biosynthesis. *J. Biol. Chem.* **276**, 33297–33300 (2001). 11435415 10.1074/jbc.C100346200

[124] Behan, R. K. & Lippard, S. J. The aging-associated enzyme CLK-1 is a member of the carboxylate-bridged diiron family of proteins. *Biochemistry* **49**, 9679–9681 (2010). 20923139 10.1021/bi101475z PMC2976817

[125] Rea, S. CLK-1/Coq7p is a DMQ mono-oxygenase and a new member of the di-iron carboxylate protein family. *FEBS Lett.* **509**, 389–394 (2001). 11749961 10.1016/s0014-5793(01)03099-x

[126] McCoy, J. G. et al. Structure of an ETHE1-like protein from *Arabidopsis thaliana*. *Acta Crystallogr. D. Biol. Crystallogr.* **62**, 964–970 (2006). 16929096 10.1107/S0907444906020592

[127] Pettinati, I. et al. Crystal structure of human persulfide dioxygenase: structural basis of ethylmalonic encephalopathy. *Hum. Mol. Genet*. **24**, 2458–2469 (2015). 25596185 10.1093/hmg/ddv007 PMC4383860

[128] Hildebrandt, T. M. & Grieshaber, M. K. Three enzymatic activities catalyze the oxidation of sulfide to thiosulfate in mammalian and invertebrate mitochondria. *FEBS J.* **275**, 3352–3361 (2008). 18494801 10.1111/j.1742-4658.2008.06482.x

[129] Sattler, S. A. et al. Characterizations of two bacterial persulfide dioxygenases of the metallo-beta-lactamase superfamily. *J. Biol. Chem.* **290**, 18914–18923 (2015). 26082492 10.1074/jbc.M115.652537 PMC4521011

[130] Maio, N. et al. Mechanisms of cellular iron sensing, regulation of erythropoiesis and mitochondrial iron utilization. *Semin. Hematol.* **58**, 161–174 (2021). 34389108 10.1053/j.seminhematol.2021.06.001 PMC8364622

[131] Weber, R. A. et al. Maintaining iron homeostasis is the key role of lysosomal acidity for cell proliferation. *Mol. Cell* **77**, 645–655.e7 (2020). 31983508 10.1016/j.molcel.2020.01.003 PMC7176020

[132] Yambire, K. F. et al. Impaired lysosomal acidification triggers iron deficiency and inflammation in vivo. *Elife* **8**, e51031 (2019). 10.7554/eLife.51031 PMC6917501 31793879

[133] Teh, M. R., Armitage, A. E. & Drakesmith, H. Why cells need iron: a compendium of iron utilisation. *Trends Endocrinol. Metab*. **35**, 1026–1049 (2024). 10.1016/j.tem.2024.04.015 PMC11616622 38760200

[134] Iwai, K. Regulation of cellular iron metabolism: Iron-dependent degradation of IRP by SCF(FBXL5) ubiquitin ligase. *Free Radic. Biol. Med.* **133**, 64–68 (2019). 30218771 10.1016/j.freeradbiomed.2018.09.011

[135] Hentze, M. W. et al. Two to tango: regulation of mammalian iron metabolism. *Cell* **142**, 24–38 (2010). 20603012 10.1016/j.cell.2010.06.028

[136] Volz, K. The functional duality of iron regulatory protein 1. *Curr. Opin. Struct. Biol.* **18**, 106–111 (2008). 18261896 10.1016/j.sbi.2007.12.010 PMC2374851

[137] Wang, H. et al. FBXL5 regulates IRP2 stability in iron homeostasis via an oxygen-responsive [2Fe2S] cluster. *Mol. Cell* **78**, 31–41 e5 (2020). 32126207 10.1016/j.molcel.2020.02.011 PMC7159994

[138] Terzi, E. M. et al. Iron-sulfur cluster deficiency can be sensed by IRP2 and regulates iron homeostasis and sensitivity to ferroptosis independent of IRP1 and FBXL5. *Sci. Adv*. **7**, eabg4302 (2021) . 10.1126/sciadv.abg4302 PMC8153722 34039609

[139] Santana-Codina, N. & Mancias, J. D. The role of NCOA4-mediated ferritinophagy in health and disease. *Pharmaceuticals* **11**, 114 (2018). 10.3390/ph11040114 PMC6316710 30360520

[140] Liu, M. Z. et al. The critical role of ferritinophagy in human disease. *Front. Pharm.* **13**, 933732 (2022). 10.3389/fphar.2022.933732 PMC9493325 36160450

[141] Mancias, J. D. et al. Quantitative proteomics identifies NCOA4 as the cargo receptor mediating ferritinophagy. *Nature* **509**, 105–109 (2014). 24695223 10.1038/nature13148 PMC4180099

[142] Santana-Codina, N., Gikandi, A. & Mancias, J. D. The role of NCOA4-mediated ferritinophagy in ferroptosis. *Adv. Exp. Med. Biol.* **1301**, 41–57 (2021). 34370287 10.1007/978-3-030-62026-4_4

[143] Kuno, S. & Iwai, K. Oxygen modulates iron homeostasis by switching iron sensing of NCOA4. *J. Biol. Chem.* **299**, 104701 (2023). 37059186 10.1016/j.jbc.2023.104701 PMC10196804

[144] Zhao, H. et al. NCOA4 requires a [3Fe-4S] to sense and maintain the iron homeostasis. *J. Biol. Chem.* **300**, 105612 (2024). 10.1016/j.jbc.2023.105612 PMC10831263 38159858

[145] Pakos-Zebrucka, K. et al. The integrated stress response. *EMBO Rep.* **17**, 1374–1395 (2016). 27629041 10.15252/embr.201642195 PMC5048378

[146] Quiros, P. M. et al. Multi-omics analysis identifies ATF4 as a key regulator of the mitochondrial stress response in mammals. *J. Cell Biol.* **216**, 2027–2045 (2017). 28566324 10.1083/jcb.201702058 PMC5496626

[147] Donnelly, N. et al. The eIF2alpha kinases: their structures and functions. *Cell Mol. Life Sci.* **70**, 3493–3511 (2013). 23354059 10.1007/s00018-012-1252-6 PMC11113696

[148] Toboz, P. et al. The amino acid sensor GCN2 controls red blood cell clearance and iron metabolism through regulation of liver macrophages. *Proc. Natl Acad. Sci. USA* **119**, e2121251119 (2022). 35994670 10.1073/pnas.2121251119 PMC9436309

[149] Wu, C. C. et al. Ribosome collisions trigger general stress responses to regulate cell fate. *Cell* **182**, 404–416.e14 (2020). 32610081 10.1016/j.cell.2020.06.006 PMC7384957

[150] Walter, P. & Ron, D. The unfolded protein response: from stress pathway to homeostatic regulation. *Science* **334**, 1081–1086 (2011). 22116877 10.1126/science.1209038

[151] Ricketts, M. D. et al. The heme-regulated inhibitor kinase requires dimerization for heme-sensing activity. *J. Biol. Chem.* **298**, 102451 (2022). 36063997 10.1016/j.jbc.2022.102451 PMC9520036

[152] Gao, P. et al. Viral evasion of PKR restriction by reprogramming cellular stress granules. *Proc. Natl Acad. Sci. USA* **119**, e2201169119 (2022). 35858300 10.1073/pnas.2201169119 PMC9303852

[153] Munir, M. & Berg, M. The multiple faces of proteinkinase R in antiviral defense. *Virulence* **4**, 85–89 (2013). 23314571 10.4161/viru.23134 PMC3544753

[154] Mick, E. et al. Distinct mitochondrial defects trigger the integrated stress response depending on the metabolic state of the cell. *Elife* **9**, e49178 (2020). 10.7554/eLife.49178 PMC7255802 32463360

[155] Guo, X. et al. Mitochondrial stress is relayed to the cytosol by an OMA1-DELE1-HRI pathway. *Nature* **579**, 427–432 (2020). 32132707 10.1038/s41586-020-2078-2 PMC7147832

[156] Fessler, E. et al. A pathway coordinated by DELE1 relays mitochondrial stress to the cytosol. *Nature* **579**, 433–437 (2020). 32132706 10.1038/s41586-020-2076-4 PMC7116715

[157] Sekine, Y. et al. A mitochondrial iron-responsive pathway regulated by DELE1. *Mol. Cell* **83**, 2059–2076.e6 (2023). 37327776 10.1016/j.molcel.2023.05.031 PMC10329284

[158] Chakrabarty, Y. et al. The HRI branch of the integrated stress response selectively triggers mitophagy. *Mol. Cell* **84**, 1090–1100.e6 (2024). 38340717 10.1016/j.molcel.2024.01.016 PMC11062084

[159] Ahola, S. et al. OMA1-mediated integrated stress response protects against ferroptosis in mitochondrial cardiomyopathy. *Cell Metab.* **34**, 1875–1891.e7 (2022). 36113464 10.1016/j.cmet.2022.08.017

[160] Dixon, S. J. et al. Ferroptosis: an iron-dependent form of nonapoptotic cell death. *Cell* **149**, 1060–1072 (2012). 22632970 10.1016/j.cell.2012.03.042 PMC3367386

[161] Levi, S. & Rovida, E. The role of iron in mitochondrial function. *Biochim. Biophys. Acta* **1790**, 629–636 (2009). 18948172 10.1016/j.bbagen.2008.09.008

[162] Gao, J. et al. Mitochondrial iron metabolism and its role in diseases. *Clin. Chim. Acta* **513**, 6–12 (2021). 33309797 10.1016/j.cca.2020.12.005

[163] Keita, M. et al. Friedreich ataxia: clinical features and new developments. *Neurodegener. Dis. Manag.* **12**, 267–283 (2022). 35766110 10.2217/nmt-2022-0011 PMC9517959

[164] Campuzano, V. et al. Friedreich’s ataxia: autosomal recessive disease caused by an intronic GAA triplet repeat expansion. *Science* **271**, 1423–1427 (1996). 8596916 10.1126/science.271.5254.1423

[165] Durr, A. et al. Clinical and genetic abnormalities in patients with Friedreich’s ataxia. *N. Engl. J. Med.* **335**, 1169–1175 (1996). 8815938 10.1056/NEJM199610173351601

[166] Tamaroff, J. et al. Friedreich’s ataxia related diabetes: epidemiology and management practice*s*. *Diab. Res. Clin. Pr.* **186**, 109828 (2022). 10.1016/j.diabres.2022.109828 PMC9075677 35301072

[167] Rummey, C. et al. Scoliosis in Friedreich’s ataxia: longitudinal characterization in a large heterogeneous cohort. *Ann. Clin. Transl. Neurol.* **8**, 1239–1250 (2021). 33949801 10.1002/acn3.51352 PMC8164850

[168] Rojas, P. et al. Neuro-ophthalmological findings in Friedreich’s ataxia. *J. Pers. Med.* 11, 708 (2021). 10.3390/jpm11080708 PMC8398238 34442352

[169] Hanson, E. et al. Heart disease in Friedreich’s ataxia. *World J. Cardiol.* **11**, 1–12 (2019). 30705738 10.4330/wjc.v11.i1.1 PMC6354072

[170] Monda, E. et al. Diagnosis and management of cardiovascular involvement in Friedreich ataxia. *Heart Fail. Clin.* **18**, 31–37 (2022). 34776081 10.1016/j.hfc.2021.07.001

[171] Lynch, D. R., Perlman, S. & Schadt, K. Omaveloxolone for the treatment of Friedreich ataxia: clinical trial results and practical considerations. *Expert Rev. Neurother.* **24**, 251–258 (2024). 38269532 10.1080/14737175.2024.2310617

[172] Subramony, S. H. & Lynch, D. L. A milestone in the treatment of ataxias: approval of omaveloxolone for Friedreich ataxia. *Cerebellum* **23**, 775–777 (2024). 37219716 10.1007/s12311-023-01568-8

[173] Ast, T. et al. Continuous, but not intermittent, regimens of hypoxia prevent and reverse ataxia in a murine model of Friedreich’s ataxia. *Hum. Mol. Genet.* **32**, 2600–2610 (2023). 37260376 10.1093/hmg/ddad091 PMC10407700

[174] Ast, T. et al. Hypoxia rescues frataxin loss by restoring iron sulfur cluster biogenesis. *Cell* **177**, 1507–1521 (2019). 31031004 10.1016/j.cell.2019.03.045 PMC6911770

[175] Chiabrando, D., Bertino, F. & Tolosano, E. Hereditary ataxia: a focus on heme metabolism and Fe-S cluster biogenesis. *Int. J. Mol. Sci.* **2**1, 3760 (2020). 10.3390/ijms21113760 PMC7312568 32466579

[176] Bekri, S. et al. Human ABC7 transporter: gene structure and mutation causing X-linked sideroblastic anemia with ataxia with disruption of cytosolic iron-sulfur protein maturation. *Blood* **96**, 3256–3264 (2000). 11050011

[177] Xiong, S. et al. The first case report of X-linked sideroblastic anemia with ataxia of Chinese origin and literature review. *Front. Pediatr.* **9**, 692459 (2021). 34354969 10.3389/fped.2021.692459 PMC8329551

[178] Pondarre, C. et al. Abcb7, the gene responsible for X-linked sideroblastic anemia with ataxia, is essential for hematopoiesis. *Blood* **109**, 3567–3569 (2007). 17192398 10.1182/blood-2006-04-015768 PMC1852240

[179] Maguire, A. et al. X-linked cerebellar ataxia and sideroblastic anaemia associated with a missense mutation in the ABC7 gene predicting V411L. *Br. J. Haematol.* **115**, 910–917 (2001). 11843825 10.1046/j.1365-2141.2001.03015.x

[180] Boultwood, J. et al. The role of the iron transporter ABCB7 in refractory anemia with ring sideroblasts. *PLoS ONE* **3**, e1970 (2008). 18398482 10.1371/journal.pone.0001970 PMC2276313

[181] Nie, Z. et al. Expression, purification and microscopic characterization of human ATP-binding cassette sub-family B member 7 protein. *Protein Expr. Purif.* **183**, 105860 (2021). 33689857 10.1016/j.pep.2021.105860

[182] Yan, Q., Shen, Y. & Yang, X. Cryo-EM structure of AMP-PNP-bound human mitochondrial ATP-binding cassette transporter ABCB7. *J. Struct. Biol.* **214**, 107832 (2022). 35041979 10.1016/j.jsb.2022.107832

[183] Camaschella, C. Iron deficiency. *Blood* **133**, 30–39 (2019). 30401704 10.1182/blood-2018-05-815944

[184] Zhang, S. et al. HRI coordinates translation necessary for protein homeostasis and mitochondrial function in erythropoiesis. *Elife* **8**, e46976 (2019). 31033440 10.7554/eLife.46976 PMC6533081

[185] Han, A. P. et al. Heme-regulated eIF2alpha kinase (HRI) is required for translational regulation and survival of erythroid precursors in iron deficiency. *EMBO J.* **20**, 6909–6918 (2001). 11726526 10.1093/emboj/20.23.6909 PMC125753

[186] Schultz, I. J. et al. Iron and porphyrin trafficking in heme biogenesis. *J. Biol. Chem.* **285**, 26753–26759 (2010). 20522548 10.1074/jbc.R110.119503 PMC2930673

[187] Morimoto, Y. et al. Azacitidine is a potential therapeutic drug for pyridoxine-refractory female X-linked sideroblastic anemia. *Blood Adv.* **6**, 1100–1114 (2022). 34781359 10.1182/bloodadvances.2021005664 PMC8864662

[188] Phillips, J. D. Heme biosynthesis and the porphyrias. *Mol. Genet. Metab.* **128**, 164–177 (2019). 31326287 10.1016/j.ymgme.2019.04.008 PMC7252266

[189] DeTure, M. A. & Dickson, D. W. The neuropathological diagnosis of Alzheimer’s disease. *Mol. Neurodegener.* **14**, 32 (2019). 31375134 10.1186/s13024-019-0333-5 PMC6679484

[190] Smith, M. A. et al. Increased iron and free radical generation in preclinical Alzheimer disease and mild cognitive impairment. *J. Alzheimers Dis.* **19**, 363–372 (2010). 20061651 10.3233/JAD-2010-1239 PMC2842004

[191] Ayton, S. et al. Cerebral quantitative susceptibility mapping predicts amyloid-beta-related cognitive decline. *Brain* **140**, 2112–2119 (2017). 28899019 10.1093/brain/awx137

[192] Diouf, I. et al. Cerebrospinal fluid ferritin levels predict brain hypometabolism in people with underlying beta-amyloid pathology. *Neurobiol. Dis.* **124**, 335–339 (2019). 30557658 10.1016/j.nbd.2018.12.010

[193] Ayton, S. et al. Ferritin levels in the cerebrospinal fluid predict Alzheimer’s disease outcomes and are regulated by APOE. *Nat. Commun.* **6**, 6760 (2015). 25988319 10.1038/ncomms7760 PMC4479012

[194] Hui, Y. et al. Long-term overexpression of heme oxygenase 1 promotes tau aggregation in mouse brain by inducing tau phosphorylation. *J. Alzheimers Dis.* **26**, 299–313 (2011). 21613741 10.3233/JAD-2011-102061

[195] Wan, W. et al. Iron deposition leads to hyperphosphorylation of tau and disruption of insulin signaling. *Front. Neurol.* **10**, 607 (2019). 31275224 10.3389/fneur.2019.00607 PMC6593079

[196] Wang, J. et al. Iron and targeted iron therapy in Alzheimer’s disease. *Int. J. Mol. Sci.* **24**, 16353 (2023). 38003544 10.3390/ijms242216353 PMC10671546

[197] Farr, A. C. & Xiong, M. P. Challenges and opportunities of deferoxamine delivery for treatment of Alzheimer’s disease, Parkinson’s disease, and intracerebral hemorrhage. *Mol. Pharm.* **18**, 593–609 (2021). 32926630 10.1021/acs.molpharmaceut.0c00474 PMC8819678

[198] Liu, G. et al. Nanoparticle-chelator conjugates as inhibitors of amyloid-beta aggregation and neurotoxicity: a novel therapeutic approach for Alzheimer disease. *Neurosci. Lett.* **455**, 187–190 (2009). 19429118 10.1016/j.neulet.2009.03.064 PMC2683427

[199] Ayton, S. et al. Deferiprone in Alzheimer disease: a randomized clinical trial. *JAMA Neurol.* e243733 (2024). 10.1001/jamaneurol.2024.3733 PMC11536302 39495531

[200] Bloem, B. R., Okun, M. S. & Klein, C. Parkinson’s disease. *Lancet* **397**, 2284–2303 (2021). 33848468 10.1016/S0140-6736(21)00218-X

[201] Engelhardt, E. & Gomes, M. D. M. Lewy and his inclusion bodies: discovery and rejection. *Dement. Neuropsychol.* **11**, 198–201 (2017). 29213511 10.1590/1980-57642016dn11-020012 PMC5710688

[202] Riederer, P. et al. Transition metals, ferritin, glutathione, and ascorbic acid in parkinsonian brains. *J. Neurochem.* **52**, 515–520 (1989). 2911028 10.1111/j.1471-4159.1989.tb09150.x

[203] Oakley, A. E. et al. Individual dopaminergic neurons show raised iron levels in Parkinson disease. *Neurology* **68**, 1820–1825 (2007). 17515544 10.1212/01.wnl.0000262033.01945.9a

[204] Dexter, D. T. et al. Increased nigral iron content and alterations in other metal ions occurring in brain in Parkinson’s disease. *J. Neurochem.* **52**, 1830–1836 (1989). 2723638 10.1111/j.1471-4159.1989.tb07264.x

[205] Gorell, J. M. et al. Increased iron-related MRI contrast in the substantia nigra in Parkinson’s disease. *Neurology* **45**, 1138–1143 (1995). p. 7783878 10.1212/wnl.45.6.1138

[206] Friedlich, A. L., Tanzi, R. E. & Rogers, J. T. The 5’-untranslated region of Parkinson’s disease alpha-synuclein messengerRNA contains a predicted iron responsive element. *Mol. Psychiatry* **12**, 222–223 (2007). 17325711 10.1038/sj.mp.4001937

[207] Bharathi, S., Indi, S. & Rao, K. S. Copper- and iron-induced differential fibril formation in alpha-synuclein: TEM study. *Neurosci. Lett.* **424**, 78–82 (2007). 17714865 10.1016/j.neulet.2007.06.052

[208] Castellani, R. J. et al. Sequestration of iron by Lewy bodies in Parkinson’s disease. *Acta Neuropathol.* **100**, 111–114 (2000). 10963356 10.1007/s004010050001

[209] Devos, D. et al. Trial of deferiprone in Parkinson’s disease. *N. Engl. J. Med.* **387**, 2045–2055 (2022). 36449420 10.1056/NEJMoa2209254

[210] Hirata, Y. et al. Lipid peroxidation increases membrane tension, Piezo1 gating, and cation permeability to execute ferroptosis. *Curr. Biol.* **33**, 1282–1294 e5 (2023). 36898371 10.1016/j.cub.2023.02.060

[211] Pedrera, L. et al. Ferroptotic pores induce Ca(2+) fluxes and ESCRT-III activation to modulate cell death kinetics. *Cell Death Differ.* **28**, 1644–1657 (2021). 33335287 10.1038/s41418-020-00691-x PMC8167089

[212] Riegman, M. et al. Ferroptosis occurs through an osmotic mechanism and propagates independently of cell rupture. *Nat. Cell Biol.* **22**, 1042–1048 (2020). 32868903 10.1038/s41556-020-0565-1 PMC7644276

[213] Friedmann Angeli, J. P. et al. Inactivation of the ferroptosis regulator Gpx4 triggers acute renal failure in mice. *Nat. Cell Biol.* **16**, 1180–1191 (2014). 25402683 10.1038/ncb3064 PMC4894846

[214] Jiang, X., Stockwell, B. R. & Conrad, M. Ferroptosis: mechanisms, biology and role in disease. *Nat. Rev. Mol. Cell Biol.* **22**, 266–282 (2021). 33495651 10.1038/s41580-020-00324-8 PMC8142022

[215] Liu, H. et al. Characterization of a patient-derived variant of GPX4 for precision therapy. *Nat. Chem. Biol.* **18**, 91–100 (2022). 34931062 10.1038/s41589-021-00915-2 PMC8712418

[216] Chen, C. et al. Ferroptosis drives photoreceptor degeneration in mice with defects in all-trans-retinal clearance. *J. Biol. Chem.* **296**, 100187 (2021). 33334878 10.1074/jbc.RA120.015779 PMC7948481

[217] Wang, H. et al. Characterization of ferroptosis in murine models of hemochromatosis. *Hepatology* **66**, 449–465 (2017). 28195347 10.1002/hep.29117 PMC5573904

[218] Battaglia, A. M. et al. Ferroptosis and cancer: mitochondria meet the “iron maiden” cell death. *Cells* **9**, 1505 (2020). 32575749 10.3390/cells9061505 PMC7349567

[219] Yuan, H. et al. CISD1 inhibits ferroptosis by protection against mitochondrial lipid peroxidation. *Biochem. Biophys. Res. Commun.* **478**, 838–844 (2016). 27510639 10.1016/j.bbrc.2016.08.034

[220] Gao, M. et al. Role of mitochondria in ferroptosis. *Mol. Cell* **73**, 354–363.e3 (2019). 30581146 10.1016/j.molcel.2018.10.042 PMC6338496

[221] Alvarez, S. W. et al. NFS1 undergoes positive selection in lung tumours and protects cells from ferroptosis. *Nature* **551**, 639–643 (2017). 29168506 10.1038/nature24637 PMC5808442

[222] Du, J. et al. Identification of Frataxin as a regulator of ferroptosis. *Redox Biol.* **32**, 101483 (2020). 32169822 10.1016/j.redox.2020.101483 PMC7068686

